# Association of Circulating, Inflammatory-Response Exosomal mRNAs With Acute Myocardial Infarction

**DOI:** 10.3389/fcvm.2021.712061

**Published:** 2021-08-19

**Authors:** Guo-dong He, Yu-qing Huang, Lin Liu, Jia-yi Huang, Kenneth Lo, Yu-ling Yu, Chao-lei Chen, Bin Zhang, Ying-qing Feng

**Affiliations:** ^1^Research Department of Medical Sciences, Guangdong Provincial People's Hospital, Guangdong Academy of Medical Sciences, Guangzhou, China; ^2^Department of Cardiology, Guangdong Cardiovascular Institute, Guangdong Provincial People's Hospital, Guangdong Academy of Medical Sciences, Guangzhou, China; ^3^Department of Epidemiology, Centre for Global Cardiometabolic Health, Brown University, Providence, RI, United States

**Keywords:** acute myocardial infarction, exosomes, mRNAs, acute inflammatory response, neutrophil, WCGNA

## Abstract

**Background:** Although many cardiovascular disease studies have focused on the microRNAs of circulating exosomes, the profile and the potential clinical diagnostic value of plasma exosomal long RNAs (exoLRs) are unknown for acute myocardial infarction (AMI).

**Methods:** In this study, the exoLR profile of 10 AMI patients, eight stable coronary artery disease (CAD) patients, and 10 healthy individuals was assessed by RNA sequencing. Bioinformatic approaches were used to investigate the characteristics and potential clinical value of exoLRs.

**Results:** Exosomal mRNAs comprised the majority of total exoLRs. Immune cell types analyzed by CIBERSORT showed that neutrophils and monocytes were significantly enriched in AMI patients, consistent with clinical baseline values. Biological process enrichment analysis and co-expression network analysis demonstrated neutrophil activation processes to be enriched in AMI patients. Furthermore, two exosomal mRNAs, *ALPL* and *CXCR2*, were identified as AMI biomarkers that may be useful for evaluation of the acute inflammatory response mediated by neutrophils.

**Conclusions:** ExoLRs were assessed in AMI patients and found to be associated with the acute inflammatory response mediated by neutrophils. Exosomal mRNAs, *ALPL* and *CXCR2*, were identified as potentially useful biomarkers for the study of AMI.

## Introduction

Exosomes secreted by most cell types are a class of lipid membrane-enclosed extracellular vesicles ranging in size from 40 to 100 nm (1, 2). These small vesicles not only contain proteins, lipids, RNAs, and metabolites of the source cell but also maintain the stability of vesicle constituents (3). Exosomes are considered crucial mediators of cell–cell communication and are promising biomarkers for disease diagnosis (4, 5).

To date, most studies have focused on examination of microRNAs in circulating exosomes with emphasis on the characterization of exosomal microRNAs associated with cardiovascular disease (4, 6, 7). However, those studies have been limited by the small quantity and specificity of available exosomal microRNAs (8).

Circular RNA (circRNA), long noncoding RNAs (lncRNA), and messenger RNA (mRNA) are long RNAs present and stabilized in exosomes (9). These exosomal RNAs may have potential functional and clinical applications (10). For example, CCL2 exosomal mRNAs derived from tubular epithelial cells and macrophages are crucial for albumin-induced tubulointerstitial inflammation (11). Indeed, two serum exosomal mRNAs, KRTAP5-4, and MAGEA3, may be potential biomarkers for the detection of colorectal cancer (12). However, very few studies have assessed the characteristics of exosomal long RNAs (exoLRs) in cardiovascular disease, e.g., AMI, which has the associated health consequences of mortality, morbidity, and monetary costs to society (13). And the profile of circulating exoLRs in AMI patients is unknown.

In this study, we explored the plasma exoLRs profiles of individuals with AMI and stable coronary artery disease (CAD), as well as healthy individuals. This was accomplished by RNA sequencing analysis, which was used to investigate the characteristics and potential clinical diagnostic value of such profiles. Plasma exoLRs profiles may not only reflect the circulating immune cell types but also distinguish patients with AMI and CAD from healthy individuals. In this manner we have identified potential biomarkers for AMI diagnosis, which may provide insight into the intrinsic basis for AMI.

## Materials and Methods

### Patients

This is a case-control study. Ten AMI, eight CAD patients, and 10 healthy individuals were recruited at the Guangdong Provincial People's Hospital from December 2018 to January 2019. The AMI and CAD patients were diagnosed by laboratory tests and coronary angiography based on the European Society of Cardiology guidelines (14, 15). Participants were 18–75 years of age and included both genders.

The healthy individuals' recruitment inclusion criteria included: normal renal and liver function; and no history of smoking, malignancy, recent cardiovascular or cerebrovascular events, rheumatologic disorders, chronic heart failure, diabetes, acute or chronic infectious disease, aortic dissection, pulmonary embolism, myocarditis, pericarditis, or congenital heart disease.

Ethical approval of human sample collection was obtained from the Ethics Committee of Guangdong Provincial People's Hospital (No. 2018160A). All patients provided informed consent.

### Extraction of Exosomal RNAs From Serum and Library Construction, Sequencing, and Data Analysis

Two milliliters of venous blood were collected into ethylene diamine tetraacetic acid (EDTA) routine blood tubes immediately at admission before coronary angiography. Plasma was separated by centrifugation at 2,000 × g for 10 min at 4°C and stored in cryogenic vials at −80°C. The exoRNeasy Serum/Plasma kit (Qiagen) was used to extract exosomal total RNA based on the manufacturer's instructions. Exosomal RNA was extracted from 1 mL of plasma. SMART technology (Clontech) was used to construct the RNA-seq libraries. RNA sequencing was performed using a illumine Nova-Seq 6000 System with the technical support of the Guangzhou Epibiotek Co., Ltd. *HISAT2* was used to align sequencing reads (16). The GENCODE database was used to annotate mRNAs and lncRNAs. CircRNAs from unmapped reads were identified with the Accurate CircRNA Finder Suite (17).

### Measurement and Characterization of Exosomes

Exosome morphological characteristics were assessed by transmission electron microscopy (TEM). Size and distribution were measured using NanoSight NS500 (NanoSight Ltd., Amesbury, United Kingdom). Exosomal protein markers CD9 and CD63 were detected by western blot.

### Immune Cells Landscape Analysis

The abundance of immune cells in serum of study groups were quantified using CIBERSOFT algorithm. The standardized gene expression data of the cells was uploaded to a publicly available online database (https://cibersort.stanford.edu/index.php). Overall, we characterized the serum distribution of 22 human immune cells based on 547 marker genes (18).

### Identification of ExoLRs and Functional Enrichment Analysis

NetworkAnalyst (http://www.networkanalyst.ca), a visual analytics platform for comprehensive gene expression profiling (19), was used to identify exoLRs that differed between AMI, CAD, and normal samples. The platform has three steps: data filtering, normalization, and difference analysis. First, exoLRs with low expression were filtered from the dataset. Variance percentile rank lower than 15% was filtered and the minimum criterion for retaining an RNA was at least 4 counts per million. Following data filtering, counts were then normalized using the trimmed mean of *M*-values normalization method. These steps reduce the influence of batch effect on experimental results. Then the “*DESeq2*” package was applied for differential analysis of count data in order to estimate variance-mean dependence in count data from high-throughput sequencing assays. Differential expression was based on a model using the negative binomial distribution. Subsequently, the data (i.e., *P*-value, fold changes, or effect sizes) were extracted. Based on this overall evidence, we identified RNAs that were significantly different in expression. RNAs with *P* < 0.01 and log_2_ fold change ≥ |1| were considered significantly different exoLRs. The “ClusterProfiler” package was used to investigate Gene Ontology (GO) enrichment analysis including biological processes, cellular components, and molecular functions (20). *P* < 0.05 was selected as the cut-off value for enriched function.

### Co-expression Networks of Exosomal mRNAs

Exosomal mRNAs were initially filtered if expression values were <1 fragment per kilobase per million (FPKM) in at least 90% of the samples. The remaining mRNAs with standard deviations >0.2 were fed into an R package for weighted correlation network analysis (21).

Appropriate soft-threshold power was selected to ensure the co-expression network based on scale-free topology. The weighted adjacencies and correlations were transformed into a topological overlap matrix (TOM), followed by calculation of the corresponding dissimilarity (1-TOM). Next, 1-TOM, as the distance measure, was applied to a hierarchical clustering analysis of genes. A dynamic tree cut algorithm was used to identify modules. Based on module eigengene and clinical trait correlations, significant modules were identified with *P* < 0.05. The top 50 exosomal mRNA connections, based on topological overlap in significant modules, were used to construct a network diagram using Cytoscape (22). Exosomal mRNAs with eigengene connectivity > 0.8, within the module, were related to clinical traits and considered candidate hub genes (23).

### Statistical Analysis

RNA expression levels are shown as means of FPKM. Comparative Venn diagrams were constructed using Venny 2.1.0 (https://bioinfogp.cnb.csic.es/tools/venny/index.html). Principal component analysis (PCA) was applied to evaluate variables within the three groups. Data were transformed into log_2_ scale and plotted using the plotPCA function in R v.3.5.2. Continuous variables that were normally distributed are displayed as means with standard deviation. One-way ANOVA followed by post-test least significant difference was performed to assess the difference among multiple groups. *P* < 0.05 was considered statistically significant. Pearson's correlation coefficient was calculated to test statistical correlation and *r* > 0.5 or *r* < −0.5 with a *P* < 0.05 was considered statistically significant. The area under the curve (AUC) of receiver operating characteristic (ROC) was applied to evaluate the specificity and sensitivity of exosomal mRNA for AMI diagnosis with 95% confidence interval (95% CI) calculated. Due to the relatively small sample size for ROC analysis, statistical power was calculated using PASS (version 15.0) with the following conditions: α = 0.05, AUC = 0.5, and *n* = 30. *P* < 0.05 was considered statistically significant. PASW Statistics 18.0 software was used for all statistical analyses.

## Results

### Clinical Baseline Characteristics of Patients

The clinical characteristics of the three groups (10 AMI, 8 CAD, and 10 controls) are presented in Table 1. Of the 10 AMI patients, nine presented with ST-elevated myocardial infarction and one with non-ST-elevated myocardial infarction. The CAD group and control groups had similar acute inflammation levels, whereas the AMI group had significantly higher levels at study entry. MB isoenzyme of creatine kinase (CKMB) and hydroxybutyrate-dehydrogenase (HBDH) were also higher in AMI patients than in the other subjects.

**Table 1:** Clinical baseline characteristics of patients.

	**AMI**	**CAD**	**CONTROL**
	***n* = 10**	***n* = 8**	***n* = 10**
**Gender**
Male	8	7	2
Female	2	1	8
Age (years)	62.80 ± 10.04^a^	53.88 ± 9.52	47.00 ± 14.02
Diabetes	4	3	0
**Hypertension**
Grade 1	1	0	0
Grade 2	1	2	0
Grade 3	2	4	0
Current smoking	3	0	0
Current drinking	2	0	0
MONO	1.01 ± 0.36^a^^,^^c^	0.55 ± 0.26	0.44 ± 0.09
MONO%	8.46 ± 3.70	8.29 ± 2.42	7.24 ± 0.78
NEUT	10.27 ± 3.62^b^^,^^d^	4.03 ± 1.80	3.96 ± 1.90
NEUT%	78.00 ± 12.72^b^^,^^d^	59.88 ± 8.01	59.58 ± 11.48
CK-MB (U/L)	235.47 ± 302.70^b^^,^^d^	11.80 ± 2.21	10.4 ± 0.64
HBDH (U/L)	699.00 ± 670.98^b^^,^^d^	89.00 ± 12.29	97.30 ± 13.21

*Results are presented as the mean ± SD. The AMI group compared to control group*,

a *P < 0.05*,

b *P < 0.01; the AMI group compared to CAD group*,

c *P < 0.05*,

d *P < 0.01; MONO, absolute monocyte count; MONO%, the percentage of monocyte; NEUT, absolute neutrophil count; NEUT%, the percentage of neutrophil; CK-MB, creatine kinase, MB Form; HBDH, hydroxybutyrate-dehydrogenase*.

### Brief Workflow for Plasma ExoLRs-Seq for Group Characterization

**Figure 1:**
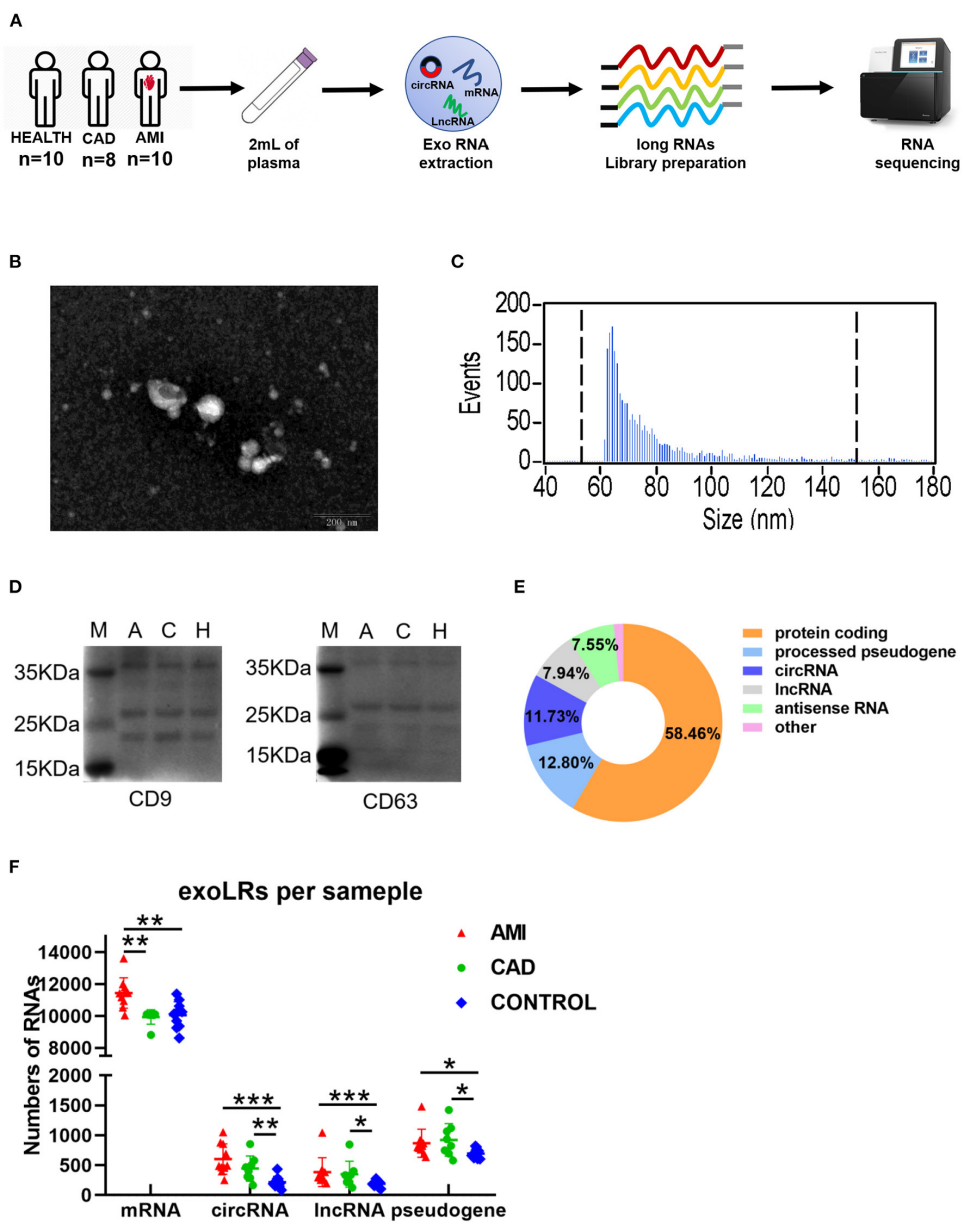
A brief view of the workflow of human plasma exosomal long RNA-seq and its characteristics in each group. **(A)** Work flow of exosomal long RNA-seq of human plasma. **(B)** Electron microscopy image of isolated exosomes. **(C)** Size distribution measurements of isolated exosomes. **(D)** Western blot analysis of exosomal markers (M, marker; A, acute myocardial infarction; C, coronary artery disease; H, healthy individuals). **(E)** Distribution of mapped reads to the genes with annotation and identified circRNA. **(F)** The number of exoLRs for each group displayed by scatterplot. Results are described as the mean ± SD (*P < 0.05, **P < 0.01, and ***P < 0.001; Exo RNA, exosomal RNA; exoLRs, exosomal long RNAs).

Reliable exoLRs-seq data were obtained by plasma isolation, purification of exosomes, exosome RNA extraction, and RNA-seq library construction (Figure 1A). TEM results showed membrane-enclosed exosome structures without similar size or uniform distribution (Figure 1B). The average diameter of the isolated exosomes was 75.83 nm measured with a NanoSight instrument (Figure 1C). Membrane markers of exosomes, CD9 and CD63, were demonstrated by Western blot (Figure 1D). mRNA constituted 58.46% of total mapped reads. Pseudogenes and circRNAs accounted for 12.80 and 11.73%, respectively, whereas lncRNAs and antisense RNAs were 7.94 and 7.55%, respectively (Figure 1E). The number of mRNAs, circRNAs, lncRNAs, and pseudogenes for the AMI group were all significantly higher than the control group (Figure 1F). Compared with the CAD group, the AMI group had a significantly higher number of mRNAs (Figure 1F).

### ExoLRs May Reflect Immune Cell Types

**Figure 2:**
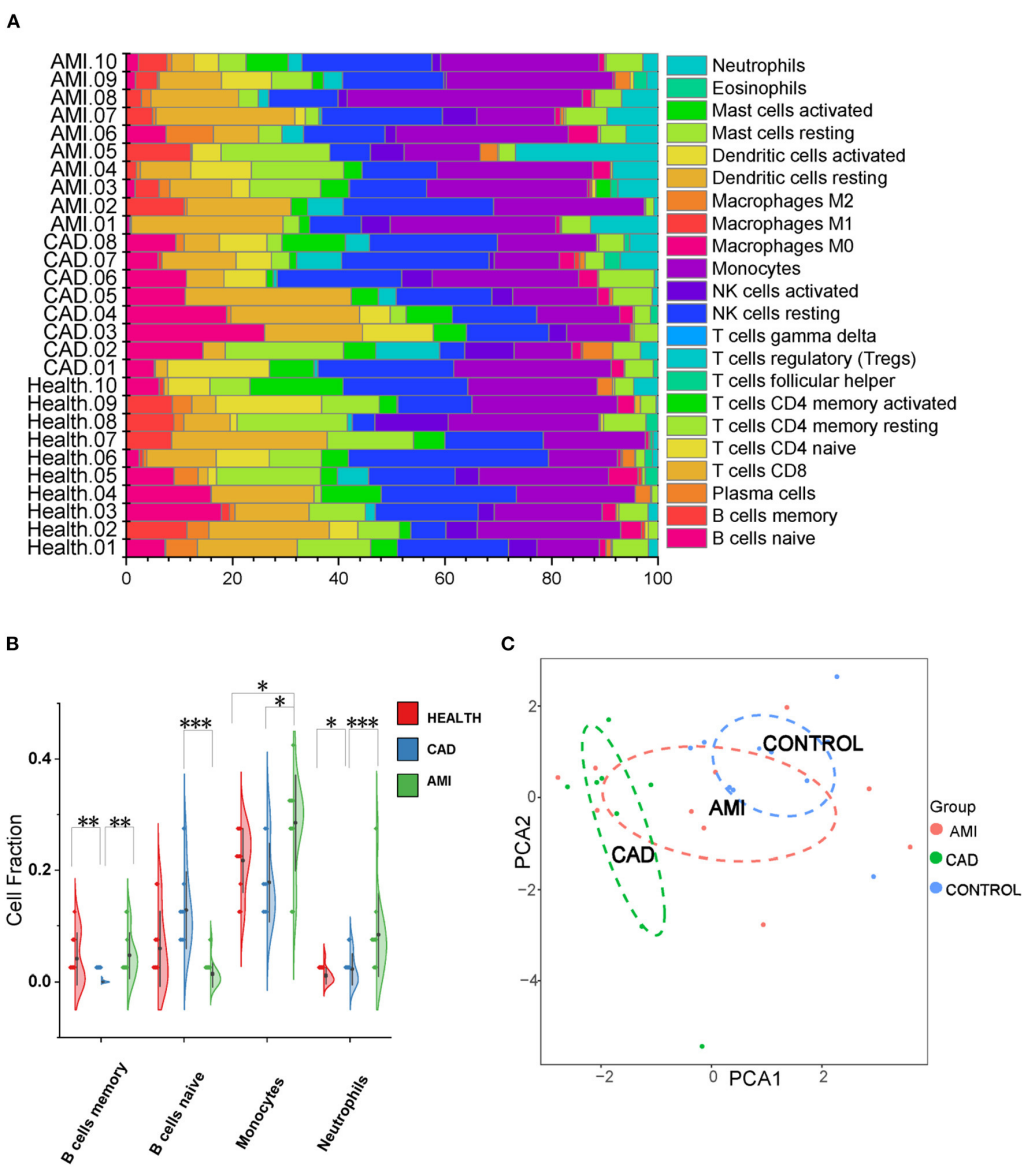
The Exosomal long RNA reflected relative fractions of different immune cell types. **(A)** Relative fraction of twenty-two types of immunocyte assessed from exosomal long RNA-seq data by CIBERSORT. **(B)** Violin plot of the fraction of four types of immune cells. Results are presented as the mean ± SD (*P < 0.05, **P < 0.01, and ***P < 0.001). **(C)** The PCA of all types of immune cells.

Twenty-two types of immune cells were assessed using the exosomal sequencing data with an established computational resource (CIBERSORT) (Figure 2A). PCA of the immunological profiles showed a non-uniform distribution (Figure 2C). Memory B cells were least in the CAD group, whereas naïve B cells of the CAD group were the highest. Neutrophils and monocytes were significantly enriched in the AMI group, which is consistent with clinical baseline characteristics (Figure 2B). Results suggest that circulating exoLRs may reflect the circulating immune cell profile.

### Comparative ExoLR Identification and Functional Enrichment Analysis of AMI and Control Groups

**Figure 3:**
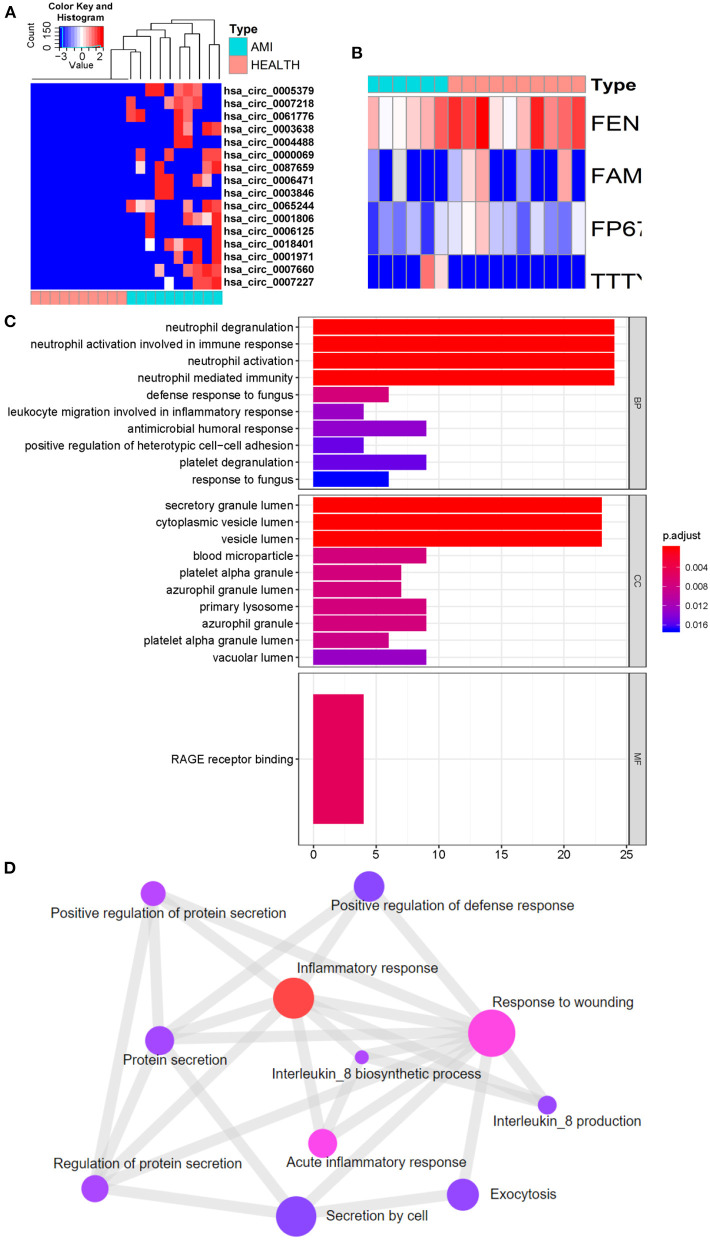
Identification of different exosomal long RNAs and functional enrichment analysis between the AMI and the control. **(A)** Heatmap of significantly circRNA between AMI and control. **(B)** Heatmap of significantly lncRNA between AMI and control. **(C,D)** Functional enrichment analysis of significantly different exosomal mRNA.

Compared with the control group, 296 different exosomal mRNAs, consisting of 254 up- and 42 down-regulated (Supplementary Table 1), were identified for the AMI group. The top 15 up and top 15 down regulated exosomal mRNAs are shown in Table 2. Only 16 different circRNAs or lncRNAs were identified (Figures 3A,B). ClusterProfiler was used to analyze and visualize functional profiles (Gene Ontology) of the 296 different exosomal mRNAs with identification of enrichment maps of biological processes by https://www.networkanalyst.ca. Results of the functional profile analysis (Supplementary Table 2) are shown as a bar plot (Figure 3C) and the enrichment map of biological processes as a network (Figure 3D). Among the top 10 biological processes in Figures 3C,D, neutrophil degranulation, neutrophil activation, and neutrophil activation involved in immune response were significantly enriched. The enrichment map (Figure 3D) showed that the inflammatory response may be the core biological process.

**Table 2:** The top 15 upregulated and top 15 downregulated exosomal mRNAs in AMI group compared with the control group.

**EntrezID**	**Gene symbol**	**log Fold-change**	***P*-value**	**Regulation**
79689	STEAP4	6.2843	8.06E-07	Upregulation
249	ALPL	6.149	4.10E-06	Upregulation
353511	PKD1P6	6.0439	6.16E-07	Upregulation
25984	KRT23	5.8028	2.45E-05	Upregulation
63926	ANKEF1	5.678	2.10E-06	Upregulation
64386	MMP25	5.6302	0.00016164	Upregulation
4286	MITF	5.603	1.43E-06	Upregulation
8653	DDX3Y	5.517	0.0095227	Upregulation
23569	PADI4	5.3678	0.00012088	Upregulation
79989	TTC26	5.2975	2.71E-05	Upregulation
4318	MMP9	5.2402	2.30E-05	Upregulation
27063	ANKRD1	5.2237	0.00038505	Upregulation
147991	DPY19L3	5.2229	0.00010934	Upregulation
84984	CEP19	5.2098	5.76E-05	Upregulation
6283	S100A12	5.1814	4.62E-05	Upregulation
642587	MIR205HG	−2.4655	0.0017053	Downregulation
387845	EEF1A1P16	−2.5355	0.00062653	Downregulation
400818	NBPF9	−2.6385	0.0069863	Downregulation
100270832	RPL5P9	−2.6572	0.0023581	Downregulation
101930105	FAM239A	−2.9793	0.0030962	Downregulation
23213	SULF1	−3.025	0.0078059	Downregulation
91695	RRP7BP	−3.2061	0.0075741	Downregulation
246	ALOX15	−3.908	0.0062638	Downregulation
253980	KCTD13	−4.0274	0.0042585	Downregulation
22982	DIP2C	−4.0735	0.0014424	Downregulation
7841	MOGS	−4.2465	0.0066529	Downregulation
27334	P2RY10	−4.2611	0.0047896	Downregulation
2582	GALE	−4.4729	0.0014293	Downregulation
7517	XRCC3	−4.5753	0.0046839	Downregulation
10518	CIB2	−4.6129	0.0005229	Downregulation

By comparison of AMI and control exoLRs, we found that circulating AMI exosomal mRNAs may play a crucial role in the acute inflammation response mediated by neutrophils.

### Comparative Exosomal mRNA Analysis of AMI and CAD Groups

**Figure 4:**
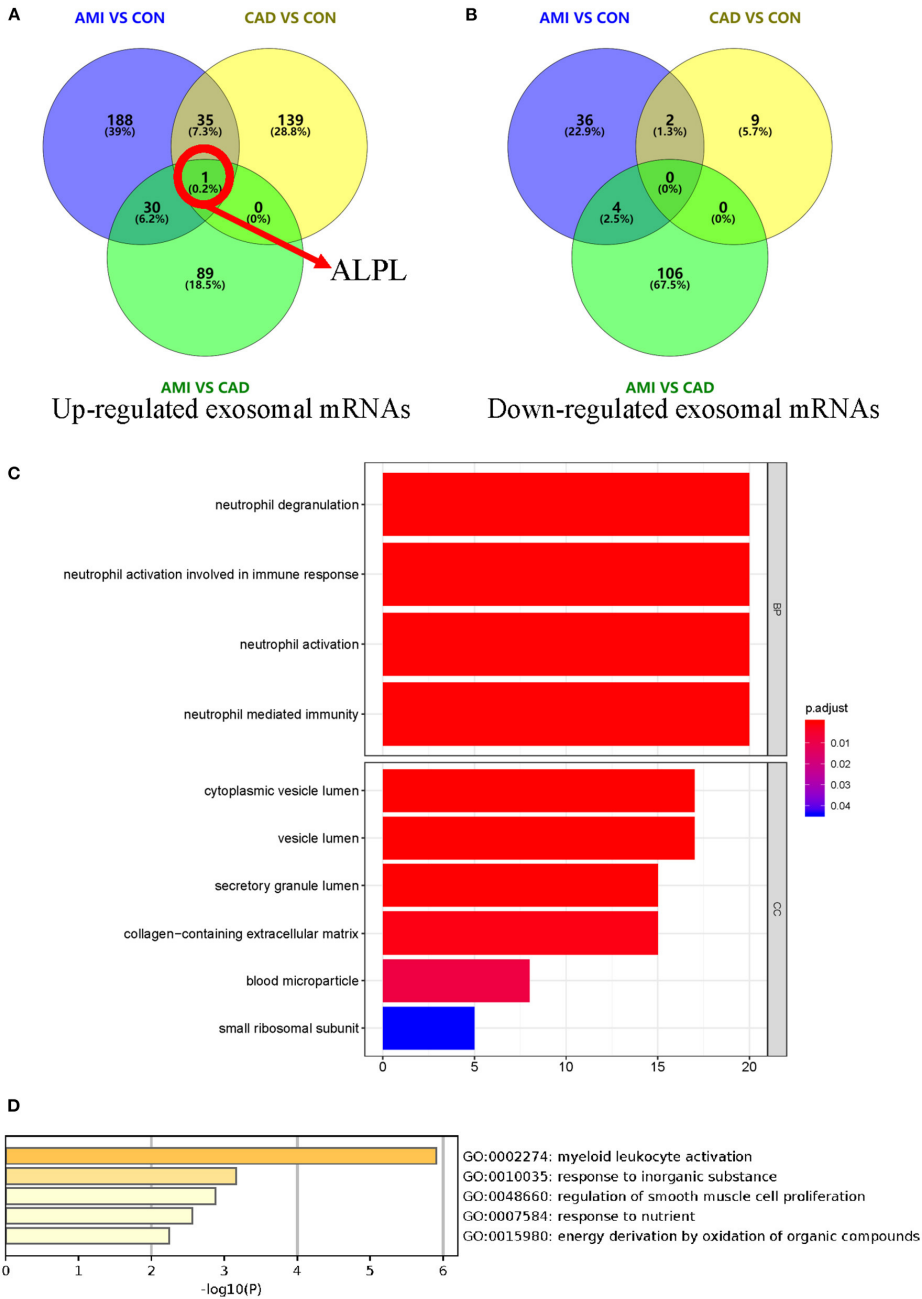
Identification of different exosomal long RNAs and functional enrichment analysis between AMI and CAD. **(A,B)** Comparation of differently up- or down-regulated exosomal mRNAs between AMI and control, AMI and CAD, and CAD and control. These three comparison sets were intersected with each other. **(C)** Functional enrichment analysis of significantly different exosomal mRNA between AMI and CAD. **(D)** Gene ontology analysis result of 35 different exosomal mRNAs between the comparison of AMI and control and comparison of AMI and CAD.

When compared with the CAD group, 230 different exosomal mRNAs (Supplementary Table 3), consisting of 120 up- and 110 down-regulated, were identified for the AMI group (Figures 4A,B). Functional profile analysis (Figure 4C and Supplementary Table 4) found neutrophil activation to play a leading role in associated biological processes. To further investigate the relationship between AMI and CAD, intersections of differently up- or down-regulated exosomal mRNAs for the AMI, CAD, and control groups are depicted in Figures 4A,B. There were 35 different exosomal mRNAs (31 up- and 4 down-regulated) that overlapped between the AMI and control groups and AMI and CAD (Supplementary Table 5). GO analysis of these 35 different exosomal mRNAs (Supplementary Table 6) found that myeloid leukocyte activation was mainly enriched (Figure 4D). *ALPL* mRNA is especially worth noting in that it was the only up-regulated exosomal mRNA among the three groups (Figure 4A) and may serve as a potential biomarker for AMI diagnosis.

### Co-expression Network Analysis of AMI Exosomal mRNAs

**Figure 5:**
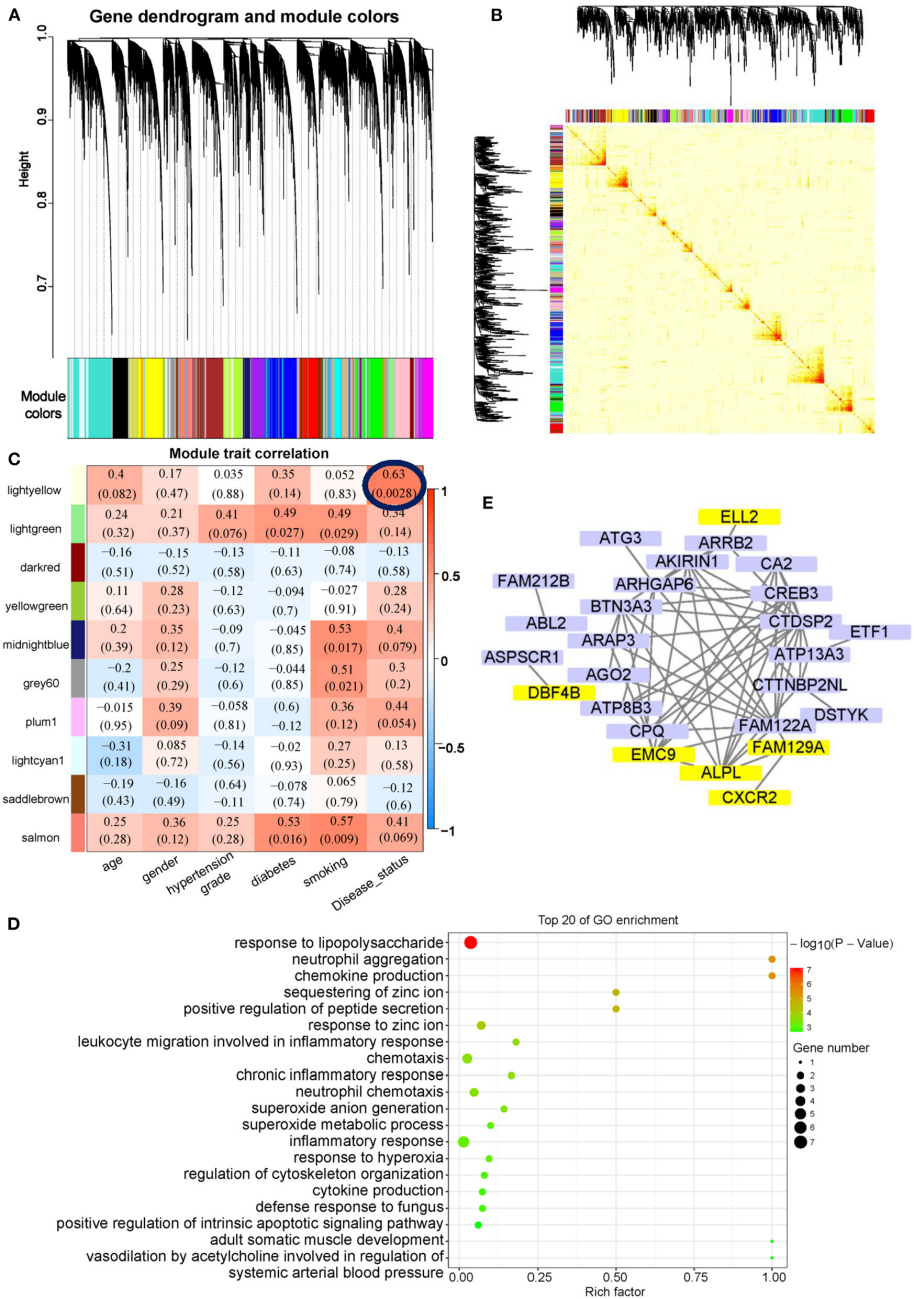
Co-expression network analysis of exosomal mRNAs in AMI. **(A)** Cluster diagram showing co-expression modules identified by WGCNA. **(B)** The heat map plot showed the topological overlap matrix (TOM) among all mRNAs. Light color shows low overlap, and red color indicates a higher overlap. The left side and the top side show the gene dendrogram and module assignment. **(C)** Heatmap of modules-trait relationship. **(D)** GO enrichment analysis of light-yellow modules. **(E)** Network visualization of the top 50 exosomal mRNAs of the topological overlap matrix in light-yellow modules. The exosomal mRNA with eigengene connectivity > 0.8 were highlighted in yellow color.

Weighted gene co-expression network analysis was used to identify key modules and highly correlated exosomal mRNAs associated with AMI. Forty-three exosomal mRNAs modules were identified by hierarchical clustering dendrogram (Figure 5A). The association of the 43 co-expression modules was analyzed by topological overlap matrix plot that consisted of the modules and the corresponding hierarchical clustering dendrogram (Figure 5B). For module-trait analyses, only the light-yellow module containing 175 exosomal mRNAs (Supplementary Table 7) was related to AMI (Figure 5C). Although there was no significantly enriched Kyoto Encyclopedia of Genes and Genomes (KEGG) biological pathways in the light-yellow module, the results of GO enrichment analysis indicated that the light-yellow module was involved in inflammation mediated by neutrophils, i.e., neutrophil aggregation and chemokine production (Figure 5D). The association between the light-yellow module and AMI was also supported by enrichment analyses of different exosomal mRNAs between AMI (Figure 3C) and control or between AMI and CAD (Figure 4D). To further analyze the core mRNA of the light-yellow module, the top 50 exosomal mRNAs of the topological overlap matrix of this module were used to construct a network diagram (Figure 5E). In this network, exosomal mRNAs (with eigengene connectivity > 0.8) in the light-yellow module were considered core mRNAs and labeled with yellow (Figure 5E). Six mRNAs including *ALPL, CXCR2, ELL2, EMC9, FAM129A*, and *DBF4B* were identified as core mRNAs.

### Potential Clinical Value of Exosomal *ALPL* and *CXCR2*

**Figure 6:**
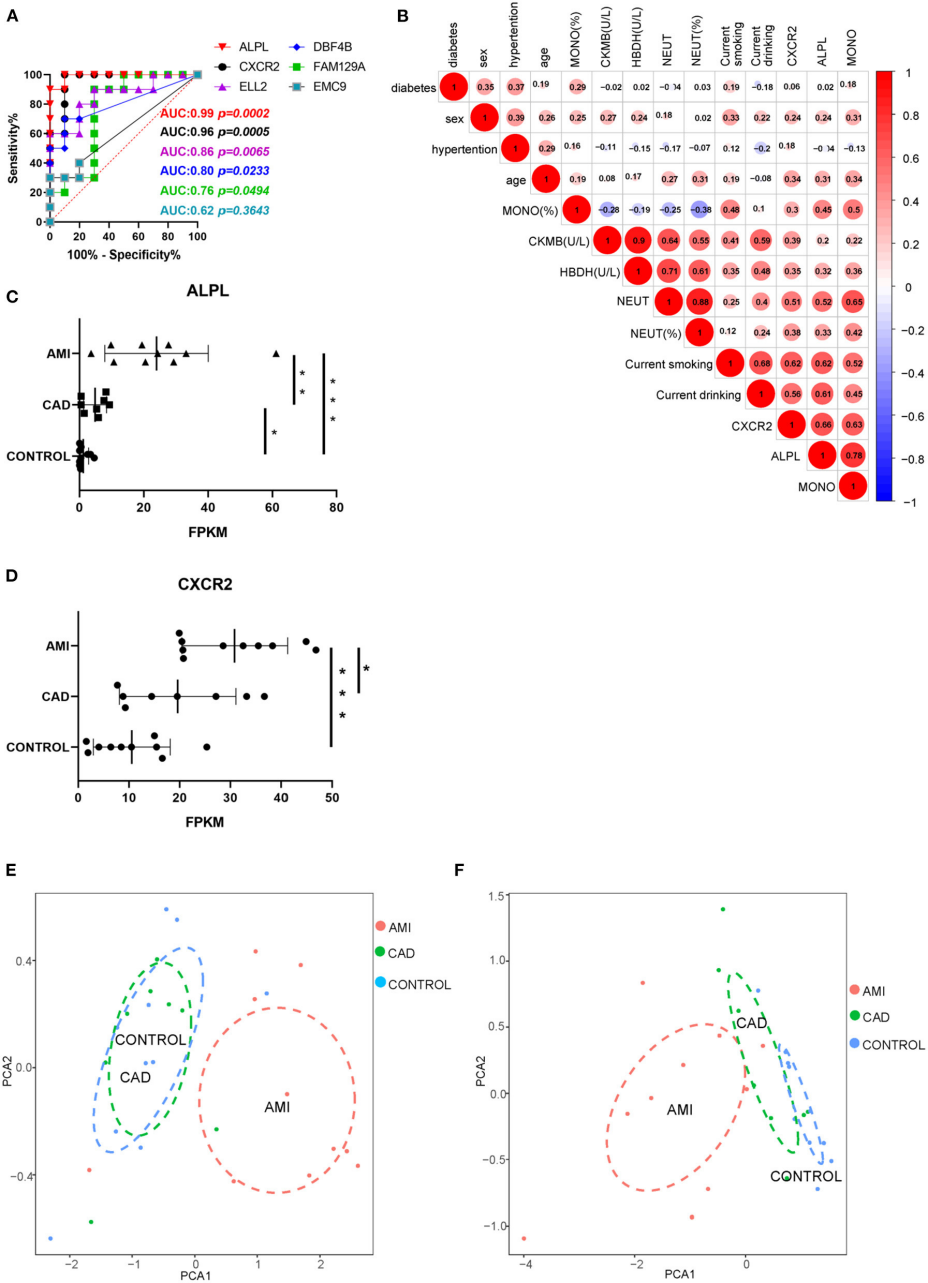
Potential clinical value of ALPL and CXCR2. **(A)** The ROC curve analysis of six core exosomal mRNAs. **(B)** The correlation analysis between these two exosomal mRNAs and clinical baseline characteristics. **(C,D)** The statistical analysis of expression of ALPL and CXCR2 among AMI, CAD, and control group. Results are presented as the mean ± SD (*P < 0.05, **P < 0.01, and ***P < 0.001). **(E)** PCA of neutrophil count and neutrophil ratio. **(F)** PCA of the expression of ALPL and CXCR2.

To estimate the potential clinical value of the six core exosomal mRNAs, ROC curve analysis was applied (Figure 6A). AUC indicated that there were two mRNAs with excellent predictive accuracy *ALPL* (AUC: 0.99, 95%CI, 0.9484–1.000, *P* = 0.0002, power = 0.99754) and *CXCR2* (AUC: 0.98, 95%CI, 0.8478–1.000, *P* = 0.0005, power = 0.68124)]. Correlational analysis of these two exosomal mRNAs and clinical baseline characteristics (Figure 6B) indicated that *ALPL* and *CXCR2* are associated with neutrophil count (*R* = 0.52 and 0.51, respectively). Statistical analysis suggested expression of exosomal mRNA *ALPL* and *CXCR2*, derived from AMI plasma, to be significantly higher than that found in the other groups (Figures 6C,D). Exosomal *ALPL* in the CAD group was also significantly higher than the control group. These results suggest that circulating exosomal *ALPL* may increase with the progression of coronary plaque to acute myocardial infarction.

Since this analysis found AMI exosomal mRNAs to be associated with acute inflammation mediated by neutrophils, we applied PCA to the neutrophil count and neutrophil ratio for the three groups. PCA of the neutrophil count and neutrophil ratio showed that although the AMI group could be separate from the other groups, there was much overlap between the CAD and control groups (Figure 6E). However, when we applied PCA to the expression of *ALPL* and *CXCR2*, the three groups could be separated (Figure 6F). In summary, exosomal *ALPL* and *CXCR2* have the predictive potential to provide greater accuracy and identification of cardiac disease.

## Discussion

Interestingly, when exosomes were initially discovered, they were considered to be waste cargo (24). However, when RNA was detected in exosomes, they were considered as promising tools for the treatment and diagnosis of diseases especially cancer (25). Long RNA species (mRNA, lncRNA, and circRNA) are found in human blood exosomes with potential clinical usefulness (26). These exosomes could serve as diagnostic markers but could also play a significant role in cell-to-cell communication by activating biological signaling pathways (27). Such exosomal RNAs could reflect and affect the progression of disease.

Exosomal microRNAs have been extensively evaluated with relation to cardiovascular disease but very few studies have assessed the features and potential clinical value of exosomal mRNAs. In this study, we found that plasma exoLRs largely consist of mRNAs. In AMI patients, alterations in these mRNAs could indicate neutrophilic inflammation of the circulatory system. Among these mRNAs, we found *ALPL* and *CXCR2* to have good predictive accuracy and may be potential biomarkers for AMI diagnosis.

The exoLRs sequencing data from this study demonstrate plasma exoLRs to be primarily mRNAs. It is not surprising that exosomal circRNAs are expressed in low abundance since they have specific spatiotemporal expression patterns (28–30). A relatively high number of long RNAs were identified in AMI samples and it is worth noting that the AMI samples in this study were from relatively aged patients. Previous studies have demonstrated aging to impact circulating extracellular vesicle concentration, size, and cargo (31, 32). Furthermore, stressors such as hypoxia, inflammation, and injury can induce cardiomyocytes or other cells to secret exosomes (33).

To provide a better understanding of exosomal mRNAs in AMI patients, we used a bioinformatic approach to compare AMI and CAD patients to healthy individuals. We also used co-expression network analysis to investigate the exosomal mRNAs of AMI patients. Results demonstrated the acute inflammatory response mediated by neutrophils to be the core biological process in AMI patients. These findings are consistent with current AMI studies. Since the cardiomyocyte is extremely sensitive to ischemic injury, reduced blood supply to the myocardium could initially cause injury and lead to an intense inflammatory response once AMI had occurred (34, 35). Furthermore, neutrophil extracellular traps have been identified as crucial triggers and structural contributors to various forms of thrombosis (36). Furthermore, neutrophilic inflammation was found to influence infarct size, healing, and cardiac function after myocardial infarction (37). Although the exact physiological role of exosome in AMI is still poorly understood, inflammation-related alterations in exosomal RNAs are associated with the biological process of AMI.

Since miscellaneous immune cells take part in cardiac repair during phases of cardiovascular disease (38), neutrophils have been traditional biomarkers (39). Although the diagnosis of acute myocardial infarction is dependent on an elevation of the serum levels of cardiac-specific troponin I, troponin T, or the myocardial band isoenzyme of creatine kinase (CK–MB), there is still a lack of biomarkers that evaluate the inflammatory response mediated by neutrophils. In this study, we found two potential biomarkers, exosomal mRNAs *ALPL* and *CXCR2*, to be specifically enriched in circulating neutrophils. Results confirmed with the Human Protein Atlas (http://www.proteinatlas.org) (40).

*ALPL* has been shown to regulate cardiac fibrosis during myocardial infarction through TGF-β1/Smads and P53 signaling pathways (41). Similarly, an *ALPL* inhibitor was found to be a potential treatment for cardiovascular disease by attenuating arterial calcification in a non-chronic kidney disease context (42). The expression level of exosomal mRNA *ALPL* in the AMI groups was the greatest, followed by CAD group, with healthy individuals having the lowest. Whether exosomal mRNA *ALPL* increases during the progression of coronary plaque to acute myocardial infarction requires further study.

*CXCR2* is an intriguing biomolecule that has cardio-protective effects and the capacity to reduce myocardial damage after myocardial ischemia-reperfusion injury (43, 44). Furthermore, *CXCR2* may play a crucial cardio-protective role in myocardial infarction through enhanced myeloid progenitor production and upregulation of cardiac adhesion molecules (37, 45). One recent study reported a process of temporal neutrophil polarization in the ischemic heart, with N1 polarized pro-inflammatory neutrophils infiltrating the heart early after AMI, while the proportion of N2 polarized anti-inflammatory neutrophils increased (46). Another interestingly recent single-cell transcriptomics study investigating temporal neutrophil diversity in the blood and heart after murine myocardial infarction indicated that all neutrophils highly expressed CXCR2, and its surface level was slightly increased in SiglecF^hi^ vs. SiglecF^low^ neutrophils at day 3 (47). Therefore, it is essential to investigate the involvement of biomarkers related to neutrophils. Our data show circulating exosomal mRNA *CXCR2* to be a potential biomarker for AMI with high diagnostic efficiency and constituted a resource for further investigation of the functional implications of neutrophils in myocardial infarction. Further studies are required to understand the mechanistic basis for secretion of exosomal mRNA *CXCR2*.

Circulating plasma exosomes are known to interact with a variety of cell types and tissue. However, the exact physiological or purpose of plasma exosomal mRNAs is poorly understood. The functionality of exosomal mRNAs depends upon whether they are intact or in fragments. In fact, some exosomal mRNAs have been found to be full length and functional (48).

Multiple limitations of the present study should be acknowledged. First, the sample size of this study was relatively small. Further studies are required that assess a larger sample size in a validated and prospective clinical trial. Second, due to the small number of patients, the effect of confounding factors, especially patients' age and gender were difficult to exclude. Third, blood samples were collected immediately at admission before coronary angiography, however, the occurred time of AMI might varied from patient to patient. This could influence the results because the composition of exosomal exoLRs would change remarkablely after AMI in a time-dependent manner. Fourth, although the result of average diameter (Figure 1C) showed that the extracellular vesicles extracted from plasma were mainly composed of exosomes, other extracellular vesicles was still contaminated during the isolation of exosomes.

In conclusion, our study explored exoLRs in AMI patients and found an association with the acute inflammatory response mediated by neutrophils. Moreover, we found that exosomal mRNAs, *ALPL* and *CXCR2*, may serve as potential useful biomarkers of the acute inflammatory response mediated by neutrophils in AMI.

## Data Availability Statement

The datasets presented in this study can be found in online repositories. The names of the repository/repositories and accession number(s) can be found below: https://www.ncbi.nlm.nih.gov/geo/ (GSE159657).

## Ethics Statement

The studies involving human participants were reviewed and approved by Guangdong Provincial People's Hospital (No. 2018160A). The patients/participants provided their written informed consent to participate in this study.

## Author Contributions

G-dH and Y-qH coordinated the testing of samples and performed the bioinformatics analyses. LL, J-yH, and KL performed the statistical analyses. Y-lY and C-lC collected the samples. BZ and Y-qF designed the original study and drafted the manuscript. All authors critically reviewed and approved the final manuscript.

## Conflict of Interest

The authors declare that the research was conducted in the absence of any commercial or financial relationships that could be construed as a potential conflict of interest.

## Publisher's Note

All claims expressed in this article are solely those of the authors and do not necessarily represent those of their affiliated organizations, or those of the publisher, the editors and the reviewers. Any product that may be evaluated in this article, or claim that may be made by its manufacturer, is not guaranteed or endorsed by the publisher.
