# Supplementary material for: Association of Circulating, Inflammatory-Response Exosomal mRNAs With Acute Myocardial Infarction
Source: Front Cardiovasc Med. 2021 Aug 19;8:712061. doi: 10.3389/fcvm.2021.712061 (PMC8418229; doi:10.3389/fcvm.2021.712061)
Supplement: Supplementary file 1 [file Table_1.DOCX]

Table S1 The different exosomal mRNAs in AMI group compared with the control group

| EntrezID | logFC | p.value | adj.p.val | symbols | name |
| --- | --- | --- | --- | --- | --- |
| 10518 | -4.6129 | 0.0005229 | 0.15 | CIB2 | calcium and integrin binding family member 2 |
| 7517 | -4.5753 | 0.0046839 | 0.30 | XRCC3 | X-ray repair cross complementing 3 |
| 2582 | -4.4729 | 0.0014293 | 0.23 | GALE | UDP-galactose-4-epimerase |
| 27334 | -4.2611 | 0.0047896 | 0.30 | P2RY10 | P2Y receptor family member 10 |
| 7841 | -4.2465 | 0.0066529 | 0.34 | MOGS | mannosyl-oligosaccharide glucosidase |
| 22982 | -4.0735 | 0.0014424 | 0.23 | DIP2C | disco interacting protein 2 homolog C |
| 253980 | -4.0274 | 0.0042585 | 0.30 | KCTD13 | potassium channel tetramerization domain containing 13 |
| 246 | -3.908 | 0.0062638 | 0.33 | ALOX15 | arachidonate 15-lipoxygenase |
| 91695 | -3.2061 | 0.0075741 | 0.34 | RRP7BP | "ribosomal RNA processing 7 homolog B, pseudogene" |
| 23213 | -3.025 | 0.0078059 | 0.34 | SULF1 | sulfatase 1 |
| 101930105 | -2.9793 | 0.0030962 | 0.28 | FAM239A | family with sequence similarity 239 member A |
| 100270832 | -2.6572 | 0.0023581 | 0.26 | RPL5P9 | ribosomal protein L5 pseudogene 9 |
| 400818 | -2.6385 | 0.0069863 | 0.34 | NBPF9 | NBPF member 9 |
| 387845 | -2.5355 | 0.00062653 | 0.15 | EEF1A1P16 | eukaryotic translation elongation factor 1 alpha 1 pseudogene 16 |
| 642587 | -2.4655 | 0.0017053 | 0.23 | MIR205HG | MIR205 host gene |
| 151579 | -2.4559 | 0.0039446 | 0.29 | BZW1P2 | basic leucine zipper and W2 domains 1 pseudogene 2 |
| 7115 | -2.435 | 0.0048852 | 0.30 | TMSB4XP1 | TMSB4X pseudogene 1 |
| 9409 | -2.1548 | 0.0036856 | 0.29 | PEX16 | peroxisomal biogenesis factor 16 |
| 729009 | -2.1506 | 0.00016434 | 0.07 | FTH1P20 | ferritin heavy chain 1 pseudogene 20 |
| 441550 | -2.0771 | 0.0075443 | 0.34 | RPS4XP11 | ribosomal protein S4X pseudogene 11 |
| 84230 | -2.0345 | 0.0094687 | 0.36 | LRRC8C | leucine rich repeat containing 8 VRAC subunit C |
| 730747 | -1.9496 | 0.0073068 | 0.34 | RPL14P3 | ribosomal protein L14 pseudogene 3 |
| 730029 | -1.7793 | 0.0051733 | 0.31 | RPSAP19 | ribosomal protein SA pseudogene 19 |
| 9695 | -1.7546 | 0.0035256 | 0.28 | EDEM1 | ER degradation enhancing alpha-mannosidase like protein 1 |
| 9117 | -1.7343 | 0.0035612 | 0.28 | SEC22C | "SEC22 homolog C, vesicle trafficking protein" |
| 100507321 | -1.7055 | 0.0019178 | 0.23 | ERVK13-1 | endogenous retrovirus group K13 member 1 |
| 106479185 | -1.7045 | 0.003613 | 0.28 | RN7SKP203 | RN7SK pseudogene 203 |
| 256364 | -1.6949 | 0.0042196 | 0.30 | EML3 | EMAP like 3 |
| 4261 | -1.6622 | 0.0056825 | 0.32 | CIITA | class II major histocompatibility complex transactivator |
| 54920 | -1.6232 | 0.0011081 | 0.20 | DUS2 | dihydrouridine synthase 2 |
| 7120 | -1.587 | 0.0018405 | 0.23 | TMSB4XP6 | TMSB4X pseudogene 6 |
| 1429 | -1.5584 | 0.0069136 | 0.34 | CRYZ | crystallin zeta |
| 5079 | -1.4847 | 0.0018422 | 0.23 | PAX5 | paired box 5 |
| 284764 | -1.4555 | 0.002776 | 0.26 | FTLP3 | ferritin light chain pseudogene 3 |
| 100873463 | -1.4294 | 0.0021707 | 0.25 | RNA5SP202 | "RNA, 5S ribosomal pseudogene 202" |
| 6086 | -1.4162 | 0.0059023 | 0.32 | RNY4 | "RNA, Ro60-associated Y4" |
| 90416 | -1.3705 | 0.0046872 | 0.30 | CCDC32 | coiled-coil domain containing 32 |
| 646531 | -1.3562 | 0.0015679 | 0.23 | YBX1P2 | Y-box binding protein 1 pseudogene 2 |
| 8740 | -1.3164 | 0.0025049 | 0.26 | TNFSF14 | TNF superfamily member 14 |
| 4585 | -1.305 | 0.003417 | 0.28 | MUC4 | "mucin 4, cell surface associated" |
| 5759 | -1.1744 | 0.0056309 | 0.32 | PTMAP2 | prothymosin alpha pseudogene 2 |
| 664709 | -1.1347 | 0.0038992 | 0.29 | HNRNPA1P10 | heterogeneous nuclear ribonucleoprotein A1 pseudogene 10 |
| 3572 | 1.0365 | 0.0051536 | 0.31 | IL6ST | interleukin 6 signal transducer |
| 6386 | 1.0423 | 0.0047413 | 0.30 | SDCBP | syndecan binding protein |
| 79627 | 1.1134 | 0.0053955 | 0.32 | OGFRL1 | opioid growth factor receptor like 1 |
| 7414 | 1.201 | 0.0081527 | 0.34 | VCL | vinculin |
| 6648 | 1.2025 | 0.0019215 | 0.23 | SOD2 | superoxide dismutase 2 |
| 5997 | 1.2635 | 0.008693 | 0.35 | RGS2 | regulator of G protein signaling 2 |
| 9554 | 1.3177 | 0.0092254 | 0.36 | SEC22B | "SEC22 homolog B, vesicle trafficking protein (gene/pseudogene)" |
| 6280 | 1.3616 | 0.00081977 | 0.17 | S100A9 | S100 calcium binding protein A9 |
| 4332 | 1.3783 | 0.0023953 | 0.26 | MNDA | myeloid cell nuclear differentiation antigen |
| 23401 | 1.4544 | 0.0032685 | 0.28 | FRAT2 | FRAT regulator of WNT signaling pathway 2 |
| 200014 | 1.5368 | 0.0089683 | 0.36 | CC2D1B | coiled-coil and C2 domain containing 1B |
| 7390 | 1.5607 | 0.0066025 | 0.34 | UROS | uroporphyrinogen III synthase |
| 54820 | 1.5626 | 0.0076126 | 0.34 | NDE1 | nudE neurodevelopment protein 1 |
| 11282 | 1.6439 | 0.0010744 | 0.19 | MGAT4B | "alpha-1,3-mannosyl-glycoprotein 4-beta-N-acetylglucosaminyltransferase B" |
| 196527 | 1.6495 | 0.0075619 | 0.34 | ANO6 | anoctamin 6 |
| 22936 | 1.6827 | 0.0073986 | 0.34 | ELL2 | elongation factor for RNA polymerase II 2 |
| 4084 | 1.7773 | 0.00063533 | 0.15 | MXD1 | MAX dimerization protein 1 |
| 5836 | 1.8589 | 0.00025725 | 0.10 | PYGL | glycogen phosphorylase L |
| 6279 | 1.9283 | 0.00020333 | 0.09 | S100A8 | S100 calcium binding protein A8 |
| 3579 | 1.9518 | 0.00087478 | 0.18 | CXCR2 | C-X-C motif chemokine receptor 2 |
| 2180 | 1.9921 | 0.00041522 | 0.12 | ACSL1 | acyl-CoA synthetase long chain family member 1 |
| 100506866 | 2.1367 | 0.0071534 | 0.34 | TTN-AS1 | TTN antisense RNA 1 |
| 10135 | 2.1375 | 1.82E-05 | 0.02 | NAMPT | nicotinamide phosphoribosyltransferase |
| 283106 | 2.14 | 0.001588 | 0.23 | CSNK2A3 | casein kinase 2 alpha 3 |
| 4535 | 2.2395 | 0.0087818 | 0.35 | MT-ND1 | mitochondrially encoded NADH dehydrogenase 1 |
| 400952 | 2.4532 | 0.0089838 | 0.36 | LINC01121 | long intergenic non-protein coding RNA 1121 |
| 10481 | 2.477 | 0.0099729 | 0.37 | HOXB13 | homeobox B13 |
| 79660 | 2.4835 | 0.008462 | 0.35 | PPP1R3B | protein phosphatase 1 regulatory subunit 3B |
| 9818 | 2.5287 | 0.0076165 | 0.34 | NUP58 | nucleoporin 58 |
| 4536 | 2.5367 | 0.0078954 | 0.34 | MT-ND2 | mitochondrially encoded NADH dehydrogenase 2 |
| 2357 | 2.5413 | 0.00038638 | 0.12 | FPR1 | formyl peptide receptor 1 |
| 23528 | 2.5782 | 0.0032417 | 0.28 | ZNF281 | zinc finger protein 281 |
| 7053 | 2.5941 | 0.0078195 | 0.34 | TGM3 | transglutaminase 3 |
| 5600 | 2.6923 | 0.0040503 | 0.29 | MAPK11 | mitogen-activated protein kinase 11 |
| 55793 | 2.7033 | 0.0082664 | 0.34 | MINDY1 | MINDY lysine 48 deubiquitinase 1 |
| 89853 | 2.7155 | 0.0039336 | 0.29 | MVB12B | multivesicular body subunit 12B |
| 23327 | 2.728 | 0.006873 | 0.34 | NEDD4L | NEDD4 like E3 ubiquitin protein ligase |
| 51768 | 2.7371 | 0.0037551 | 0.29 | TM7SF3 | transmembrane 7 superfamily member 3 |
| 10280 | 2.7945 | 0.0099033 | 0.37 | SIGMAR1 | sigma non-opioid intracellular receptor 1 |
| 408050 | 2.8292 | 0.0017029 | 0.23 | NOMO3 | NODAL modulator 3 |
| 978 | 2.8322 | 0.0095161 | 0.36 | CDA | cytidine deaminase |
| 3734 | 2.8713 | 0.0078619 | 0.34 | ANOS2P | "anosmin 2, pseudogene" |
| 55771 | 2.897 | 0.003755 | 0.29 | PRR11 | proline rich 11 |
| 317762 | 2.9153 | 0.0052197 | 0.31 | CCDC85C | coiled-coil domain containing 85C |
| 7004 | 2.9672 | 0.0095512 | 0.36 | TEAD4 | TEA domain transcription factor 4 |
| 54885 | 2.9689 | 0.0044532 | 0.30 | TBC1D8B | TBC1 domain family member 8B |
| 3783 | 2.9999 | 0.0026929 | 0.26 | KCNN4 | potassium calcium-activated channel subfamily N member 4 |
| 7518 | 3.0167 | 0.0088437 | 0.35 | XRCC4 | X-ray repair cross complementing 4 |
| 51660 | 3.0381 | 0.0099856 | 0.37 | MPC1 | mitochondrial pyruvate carrier 1 |
| 7139 | 3.0387 | 0.0053588 | 0.32 | TNNT2 | "troponin T2, cardiac type" |
| 646309 | 3.0548 | 0.0026071 | 0.26 | NAMPTP1 | nicotinamide phosphoribosyltransferase pseudogene 1 |
| 57413 | 3.0874 | 0.0077802 | 0.34 | TMIGD3 | transmembrane and immunoglobulin domain containing 3 |
| 23544 | 3.1261 | 0.0057226 | 0.32 | SEZ6L | seizure related 6 homolog like |
| 23483 | 3.1576 | 0.0094448 | 0.36 | TGDS | "TDP-glucose 4,6-dehydratase" |
| 100131691 | 3.1706 | 0.0031257 | 0.28 | MZF1-AS1 | MZF1 antisense RNA 1 |
| 57405 | 3.1711 | 0.0083685 | 0.35 | SPC25 | SPC25 component of NDC80 kinetochore complex |
| 57463 | 3.196 | 0.0091668 | 0.36 | AMIGO1 | adhesion molecule with Ig like domain 1 |
| 3674 | 3.1968 | 0.0076974 | 0.34 | ITGA2B | integrin subunit alpha 2b |
| 63932 | 3.2218 | 0.007425 | 0.34 | CXorf56 | chromosome X open reading frame 56 |
| 83869 | 3.2293 | 0.0084835 | 0.35 | TTTY14 | "testis-specific transcript, Y-linked 14" |
| 336 | 3.2324 | 0.0066806 | 0.34 | APOA2 | apolipoprotein A2 |
| 158431 | 3.2474 | 0.0074212 | 0.34 | ZNF782 | zinc finger protein 782 |
| 7038 | 3.2565 | 0.0032674 | 0.28 | TG | thyroglobulin |
| 5241 | 3.2568 | 0.0071621 | 0.34 | PGR | progesterone receptor |
| 9379 | 3.2616 | 0.0091148 | 0.36 | NRXN2 | neurexin 2 |
| 1991 | 3.2658 | 0.0036367 | 0.28 | ELANE | "elastase, neutrophil expressed" |
| 202333 | 3.2724 | 0.0054822 | 0.32 | CMYA5 | cardiomyopathy associated 5 |
| 65998 | 3.2892 | 0.0097567 | 0.36 | C11orf95 | chromosome 11 open reading frame 95 |
| 1728 | 3.2933 | 0.0065101 | 0.34 | NQO1 | NAD(P)H quinone dehydrogenase 1 |
| 64928 | 3.3015 | 0.0063708 | 0.34 | MRPL14 | mitochondrial ribosomal protein L14 |
| 140739 | 3.3016 | 0.0033046 | 0.28 | UBE2F | ubiquitin conjugating enzyme E2 F (putative) |
| 3490 | 3.3081 | 0.0082444 | 0.34 | IGFBP7 | insulin like growth factor binding protein 7 |
| 8974 | 3.3084 | 0.0012069 | 0.21 | P4HA2 | prolyl 4-hydroxylase subunit alpha 2 |
| 654 | 3.3094 | 0.0039039 | 0.29 | BMP6 | bone morphogenetic protein 6 |
| 22911 | 3.311 | 0.0088006 | 0.35 | WDR47 | WD repeat domain 47 |
| 83540 | 3.314 | 0.008114 | 0.34 | NUF2 | NUF2 component of NDC80 kinetochore complex |
| 8310 | 3.3382 | 0.0099734 | 0.37 | ACOX3 | "acyl-CoA oxidase 3, pristanoyl" |
| 4163 | 3.3386 | 0.0046574 | 0.30 | MCC | MCC regulator of WNT signaling pathway |
| 56063 | 3.3499 | 0.002746 | 0.26 | TMEM234 | transmembrane protein 234 |
| 7728 | 3.3572 | 0.0027866 | 0.26 | ZNF175 | zinc finger protein 175 |
| 7620 | 3.3702 | 0.0029657 | 0.27 | ZNF69 | zinc finger protein 69 |
| 7100 | 3.3719 | 0.0082124 | 0.34 | TLR5 | toll like receptor 5 |
| 6877 | 3.3803 | 0.0095844 | 0.36 | TAF5 | TATA-box binding protein associated factor 5 |
| 84321 | 3.3886 | 0.0064198 | 0.34 | THOC3 | THO complex 3 |
| 344558 | 3.3944 | 0.0053759 | 0.32 | SH3RF3 | SH3 domain containing ring finger 3 |
| 153562 | 3.3944 | 0.0096789 | 0.36 | MARVELD2 | MARVEL domain containing 2 |
| 51097 | 3.4032 | 0.0052167 | 0.31 | SCCPDH | saccharopine dehydrogenase (putative) |
| 54751 | 3.4143 | 0.0068823 | 0.34 | FBLIM1 | filamin binding LIM protein 1 |
| 9666 | 3.4171 | 0.0059591 | 0.32 | DZIP3 | DAZ interacting zinc finger protein 3 |
| 54805 | 3.4328 | 0.0085718 | 0.35 | CNNM2 | cyclin and CBS domain divalent metal cation transport mediator 2 |
| 100506649 | 3.4566 | 0.0080169 | 0.34 | PXN-AS1 | PXN antisense RNA 1 |
| 79968 | 3.4595 | 0.0060667 | 0.33 | WDR76 | WD repeat domain 76 |
| 79064 | 3.4651 | 0.0082827 | 0.34 | TMEM223 | transmembrane protein 223 |
| 57171 | 3.4695 | 0.0072844 | 0.34 | DOLPP1 | dolichyldiphosphatase 1 |
| 8635 | 3.4936 | 0.0015255 | 0.23 | RNASET2 | ribonuclease T2 |
| 2996 | 3.5071 | 0.006851 | 0.34 | GYPE | glycophorin E (MNS blood group) |
| 115827 | 3.515 | 0.0075346 | 0.34 | RAB3C | "RAB3C, member RAS oncogene family" |
| 11096 | 3.5168 | 0.0031402 | 0.28 | ADAMTS5 | ADAM metallopeptidase with thrombospondin type 1 motif 5 |
| 1466 | 3.5197 | 0.0092532 | 0.36 | CSRP2 | cysteine and glycine rich protein 2 |
| 285965 | 3.5215 | 0.006057 | 0.33 | EPHA1-AS1 | EPHA1 antisense RNA 1 |
| 2622 | 3.5216 | 0.0021237 | 0.25 | GAS8 | growth arrest specific 8 |
| 27034 | 3.5243 | 0.0096799 | 0.36 | ACAD8 | acyl-CoA dehydrogenase family member 8 |
| 100506881 | 3.5269 | 0.0025472 | 0.26 | MKLN1-AS | MKLN1 antisense RNA |
| 3995 | 3.5379 | 0.0073557 | 0.34 | FADS3 | fatty acid desaturase 3 |
| 221710 | 3.5444 | 0.0018117 | 0.23 | SMIM13 | small integral membrane protein 13 |
| 51053 | 3.5552 | 0.0040648 | 0.29 | GMNN | geminin DNA replication inhibitor |
| 100131825 | 3.56 | 0.0074234 | 0.34 | CADM3-AS1 | CADM3 antisense RNA 1 |
| 2244 | 3.5605 | 0.0086709 | 0.35 | FGB | fibrinogen beta chain |
| 4008 | 3.5606 | 0.0034655 | 0.28 | LMO7 | LIM domain 7 |
| 85413 | 3.5622 | 0.0094853 | 0.36 | SLC22A16 | solute carrier family 22 member 16 |
| 284348 | 3.5711 | 0.0070843 | 0.34 | LYPD5 | LY6/PLAUR domain containing 5 |
| 7422 | 3.5721 | 0.0079437 | 0.34 | VEGFA | vascular endothelial growth factor A |
| 22915 | 3.5753 | 0.0080117 | 0.34 | MMRN1 | multimerin 1 |
| 64105 | 3.5813 | 0.0033202 | 0.28 | CENPK | centromere protein K |
| 64753 | 3.5843 | 0.0094059 | 0.36 | CCDC136 | coiled-coil domain containing 136 |
| 147645 | 3.5854 | 0.0017024 | 0.23 | VSIG10L | V-set and immunoglobulin domain containing 10 like |
| 81669 | 3.5856 | 0.0089555 | 0.36 | CCNL2 | cyclin L2 |
| 388567 | 3.5921 | 0.0013092 | 0.22 | ZNF749 | zinc finger protein 749 |
| 22932 | 3.5974 | 0.0054323 | 0.32 | POMZP3 | POM121 and ZP3 fusion |
| 64108 | 3.5985 | 0.0031654 | 0.28 | RTP4 | receptor transporter protein 4 |
| 5046 | 3.6014 | 0.0048608 | 0.30 | PCSK6 | proprotein convertase subtilisin/kexin type 6 |
| 84519 | 3.6044 | 0.00088798 | 0.18 | ACRBP | acrosin binding protein |
| 84895 | 3.6086 | 0.0048208 | 0.30 | MIGA2 | mitoguardin 2 |
| 10810 | 3.6103 | 0.0047039 | 0.30 | WASF3 | WASP family member 3 |
| 23768 | 3.6205 | 0.0075166 | 0.34 | FLRT2 | fibronectin leucine rich transmembrane protein 2 |
| 229 | 3.6336 | 0.0045172 | 0.30 | ALDOB | "aldolase, fructose-bisphosphate B" |
| 90326 | 3.6388 | 0.004472 | 0.30 | THAP3 | THAP domain containing 3 |
| 7263 | 3.6392 | 0.0080895 | 0.34 | TST | thiosulfate sulfurtransferase |
| 9203 | 3.6428 | 0.0079727 | 0.34 | ZMYM3 | zinc finger MYM-type containing 3 |
| 4647 | 3.6443 | 0.0085067 | 0.35 | MYO7A | myosin VIIA |
| 1241 | 3.6676 | 0.004513 | 0.30 | LTB4R | leukotriene B4 receptor |
| 201191 | 3.675 | 0.0058197 | 0.32 | SAMD14 | sterile alpha motif domain containing 14 |
| 79154 | 3.6914 | 0.00089446 | 0.18 | DHRS11 | dehydrogenase/reductase 11 |
| 23139 | 3.6946 | 0.0017071 | 0.23 | MAST2 | microtubule associated serine/threonine kinase 2 |
| 91869 | 3.7025 | 0.00055832 | 0.15 | RFT1 | RFT1 homolog |
| 4796 | 3.7134 | 0.0075484 | 0.34 | TONSL | "tonsoku like, DNA repair protein" |
| 64925 | 3.7144 | 0.0069558 | 0.34 | CCDC71 | coiled-coil domain containing 71 |
| 6253 | 3.7255 | 0.0056301 | 0.32 | RTN2 | reticulon 2 |
| 10040 | 3.7292 | 0.00072698 | 0.16 | TOM1L1 | target of myb1 like 1 membrane trafficking protein |
| 100506668 | 3.7359 | 0.0068474 | 0.34 | NRAV | negative regulator of antiviral response |
| 8654 | 3.7374 | 0.0032702 | 0.28 | PDE5A | phosphodiesterase 5A |
| 8438 | 3.7385 | 0.0019814 | 0.24 | RAD54L | RAD54 like |
| 9468 | 3.7406 | 0.00968 | 0.36 | PCYT1B | "phosphate cytidylyltransferase 1, choline, beta" |
| 23322 | 3.7543 | 0.0044876 | 0.30 | RPGRIP1L | RPGRIP1 like |
| 79922 | 3.7544 | 0.0022158 | 0.25 | MRM1 | mitochondrial rRNA methyltransferase 1 |
| 55287 | 3.7618 | 0.0028809 | 0.27 | TMEM40 | transmembrane protein 40 |
| 1668 | 3.7662 | 0.0055309 | 0.32 | DEFA3 | defensin alpha 3 |
| 163255 | 3.772 | 0.0014161 | 0.23 | ZNF540 | zinc finger protein 540 |
| 3485 | 3.7751 | 0.0064281 | 0.34 | IGFBP2 | insulin like growth factor binding protein 2 |
| 80216 | 3.7858 | 0.004719 | 0.30 | ALPK1 | alpha kinase 1 |
| 2204 | 3.7871 | 0.0019083 | 0.23 | FCAR | Fc fragment of IgA receptor |
| 8996 | 3.7985 | 0.0043995 | 0.30 | NOL3 | nucleolar protein 3 |
| 10733 | 3.799 | 0.0024506 | 0.26 | PLK4 | polo like kinase 4 |
| 79943 | 3.8035 | 0.0048661 | 0.30 | ZNF696 | zinc finger protein 696 |
| 91156 | 3.8089 | 0.000927 | 0.18 | IGFN1 | immunoglobulin like and fibronectin type III domain containing 1 |
| 5818 | 3.8153 | 0.0044851 | 0.30 | NECTIN1 | nectin cell adhesion molecule 1 |
| 80010 | 3.8193 | 0.002458 | 0.26 | RMI1 | RecQ mediated genome instability 1 |
| 283316 | 3.8208 | 0.0025789 | 0.26 | CD163L1 | CD163 molecule like 1 |
| 8814 | 3.8382 | 0.0039935 | 0.29 | CDKL1 | cyclin dependent kinase like 1 |
| 57573 | 3.8461 | 0.003613 | 0.28 | ZNF471 | zinc finger protein 471 |
| 51527 | 3.8483 | 0.0058879 | 0.32 | GSKIP | GSK3B interacting protein |
| 140838 | 3.8491 | 0.0066599 | 0.34 | NANP | N-acetylneuraminic acid phosphatase |
| 1962 | 3.8547 | 0.0062101 | 0.33 | EHHADH | enoyl-CoA hydratase and 3-hydroxyacyl CoA dehydrogenase |
| 1439 | 3.8772 | 0.0023051 | 0.26 | CSF2RB | colony stimulating factor 2 receptor subunit beta |
| 64927 | 3.8775 | 0.002588 | 0.26 | TTC23 | tetratricopeptide repeat domain 23 |
| 79887 | 3.8778 | 0.0058743 | 0.32 | PLBD1 | phospholipase B domain containing 1 |
| 4702 | 3.8815 | 0.0023981 | 0.26 | NDUFA8 | NADH:ubiquinone oxidoreductase subunit A8 |
| 80174 | 3.9192 | 0.0045522 | 0.30 | DBF4B | DBF4 zinc finger B |
| 401022 | 3.9419 | 0.0025353 | 0.26 | HAGLR | HOXD antisense growth-associated long non-coding RNA |
| 79990 | 3.9434 | 0.0072398 | 0.34 | PLEKHH3 | "pleckstrin homology, MyTH4 and FERM domain containing H3" |
| 5831 | 3.9685 | 0.0081899 | 0.34 | PYCR1 | pyrroline-5-carboxylate reductase 1 |
| 6374 | 3.9718 | 0.0077372 | 0.34 | CXCL5 | C-X-C motif chemokine ligand 5 |
| 284252 | 3.9782 | 0.0022502 | 0.25 | KCTD1 | potassium channel tetramerization domain containing 1 |
| 57648 | 3.9797 | 0.0052344 | 0.31 | KIAA1522 | KIAA1522 |
| 4648 | 3.9829 | 0.0048168 | 0.30 | MYO7B | myosin VIIB |
| 6948 | 4.011 | 0.0043469 | 0.30 | TCN2 | transcobalamin 2 |
| 5794 | 4.0115 | 0.0018034 | 0.23 | PTPRH | protein tyrosine phosphatase receptor type H |
| 7850 | 4.0144 | 0.0071425 | 0.34 | IL1R2 | interleukin 1 receptor type 2 |
| 83667 | 4.0221 | 0.0021516 | 0.25 | SESN2 | sestrin 2 |
| 23155 | 4.0251 | 0.0095896 | 0.36 | CLCC1 | chloride channel CLIC like 1 |
| 388753 | 4.0586 | 0.0023754 | 0.26 | COA6 | cytochrome c oxidase assembly factor 6 |
| 64375 | 4.0677 | 0.0079817 | 0.34 | IKZF4 | IKAROS family zinc finger 4 |
| 5616 | 4.086 | 0.0056075 | 0.32 | PRKY | protein kinase Y-linked (pseudogene) |
| 8876 | 4.1218 | 0.001211 | 0.21 | VNN1 | vanin 1 |
| 54957 | 4.1365 | 0.0016184 | 0.23 | TXNL4B | thioredoxin like 4B |
| 54210 | 4.1492 | 0.0027275 | 0.26 | TREM1 | triggering receptor expressed on myeloid cells 1 |
| 1201 | 4.151 | 0.0016626 | 0.23 | CLN3 | "CLN3 lysosomal/endosomal transmembrane protein, battenin" |
| 4998 | 4.1515 | 0.0035702 | 0.28 | ORC1 | origin recognition complex subunit 1 |
| 4353 | 4.162 | 0.0052803 | 0.32 | MPO | myeloperoxidase |
| 144423 | 4.1692 | 0.0020854 | 0.25 | GLT1D1 | glycosyltransferase 1 domain containing 1 |
| 8632 | 4.1697 | 0.00040575 | 0.12 | DNAH17 | dynein axonemal heavy chain 17 |
| 5893 | 4.1715 | 0.0032811 | 0.28 | RAD52 | "RAD52 homolog, DNA repair protein" |
| 4013 | 4.174 | 0.00042009 | 0.12 | VWA5A | von Willebrand factor A domain containing 5A |
| 1401 | 4.1821 | 0.00076596 | 0.17 | CRP | C-reactive protein |
| 1009 | 4.2039 | 0.0057777 | 0.32 | CDH11 | cadherin 11 |
| 152195 | 4.2136 | 0.0035994 | 0.28 | NUDT16P1 | nudix hydrolase 16 pseudogene 1 |
| 343990 | 4.2376 | 0.0056771 | 0.32 | KIAA1211L | KIAA1211 like |
| 55217 | 4.2386 | 0.0009101 | 0.18 | TMLHE | "trimethyllysine hydroxylase, epsilon" |
| 50486 | 4.2431 | 0.00056764 | 0.15 | G0S2 | G0/G1 switch 2 |
| 125 | 4.2543 | 0.00055812 | 0.15 | ADH1B | "alcohol dehydrogenase 1B (class I), beta polypeptide" |
| 144132 | 4.2597 | 0.0018373 | 0.23 | DNHD1 | dynein heavy chain domain 1 |
| 23604 | 4.2659 | 0.0015949 | 0.23 | DAPK2 | death associated protein kinase 2 |
| 440145 | 4.2783 | 0.00284 | 0.27 | MZT1 | mitotic spindle organizing protein 1 |
| 91662 | 4.2919 | 0.0040718 | 0.29 | NLRP12 | NLR family pyrin domain containing 12 |
| 10112 | 4.2929 | 0.0038868 | 0.29 | KIF20A | kinesin family member 20A |
| 79022 | 4.3195 | 0.0061532 | 0.33 | TMEM106C | transmembrane protein 106C |
| 83716 | 4.3331 | 0.0010424 | 0.19 | CRISPLD2 | cysteine rich secretory protein LCCL domain containing 2 |
| 7768 | 4.349 | 0.001074 | 0.19 | ZNF225 | zinc finger protein 225 |
| 2628 | 4.3561 | 0.00028478 | 0.11 | GATM | glycine amidinotransferase |
| 64089 | 4.3729 | 0.00065032 | 0.15 | SNX16 | sorting nexin 16 |
| 4571 | 4.4006 | 0.007766 | 0.34 | MT-TP | mitochondrially encoded tRNA proline |
| 284716 | 4.4043 | 0.00049297 | 0.14 | RIMKLA | ribosomal modification protein rimK like family member A |
| 8794 | 4.4198 | 0.0019588 | 0.24 | TNFRSF10C | TNF receptor superfamily member 10c |
| 119032 | 4.4953 | 0.0015429 | 0.23 | BORCS7 | BLOC-1 related complex subunit 7 |
| 2266 | 4.4954 | 4.45E-05 | 0.04 | FGG | fibrinogen gamma chain |
| 4578 | 4.505 | 0.0043747 | 0.30 | MT-TW | mitochondrially encoded tRNA tryptophan |
| 929 | 4.5285 | 0.0039328 | 0.29 | CD14 | CD14 molecule |
| 8541 | 4.5349 | 0.0006054 | 0.15 | PPFIA3 | PTPRF interacting protein alpha 3 |
| 8642 | 4.5995 | 0.0015652 | 0.23 | DCHS1 | dachsous cadherin-related 1 |
| 11240 | 4.6291 | 0.00040794 | 0.12 | PADI2 | peptidyl arginine deiminase 2 |
| 8287 | 4.6764 | 0.0093457 | 0.36 | USP9Y | ubiquitin specific peptidase 9 Y-linked |
| 7018 | 4.7498 | 2.27E-05 | 0.02 | TF | transferrin |
| 80763 | 4.7834 | 0.0010414 | 0.19 | SPX | spexin hormone |
| 3240 | 4.8001 | 8.20E-05 | 0.05 | HP | haptoglobin |
| 285382 | 4.8813 | 0.0001095 | 0.06 | C3orf70 | chromosome 3 open reading frame 70 |
| 8705 | 4.8937 | 0.00065635 | 0.15 | B3GALT4 | "beta-1,3-galactosyltransferase 4" |
| 56833 | 4.8975 | 6.94E-05 | 0.05 | SLAMF8 | SLAM family member 8 |
| 5724 | 4.8984 | 0.00059848 | 0.15 | PTAFR | platelet activating factor receptor |
| 23306 | 4.9249 | 0.00022518 | 0.09 | NEMP1 | nuclear envelope integral membrane protein 1 |
| 8993 | 4.9615 | 0.00030858 | 0.12 | PGLYRP1 | peptidoglycan recognition protein 1 |
| 8273 | 4.9937 | 0.00013496 | 0.07 | SLC10A3 | solute carrier family 10 member 3 |
| 9625 | 4.9998 | 0.0015601 | 0.23 | AATK | apoptosis associated tyrosine kinase |
| 84969 | 5.001 | 0.0013972 | 0.23 | TOX2 | TOX high mobility group box family member 2 |
| 51188 | 5.0232 | 0.00015392 | 0.07 | SS18L2 | SS18 like 2 |
| 55471 | 5.062 | 4.77E-06 | 0.01 | NDUFAF7 | NADH:ubiquinone oxidoreductase complex assembly factor 7 |
| 7404 | 5.0667 | 0.0033213 | 0.28 | UTY | "ubiquitously transcribed tetratricopeptide repeat containing, Y-linked" |
| 2243 | 5.095 | 3.39E-06 | 0.01 | FGA | fibrinogen alpha chain |
| 9853 | 5.1283 | 0.00034797 | 0.12 | RUSC2 | RUN and SH3 domain containing 2 |
| 246126 | 5.1656 | 0.000412 | 0.12 | TXLNGY | "taxilin gamma pseudogene, Y-linked" |
| 6283 | 5.1814 | 4.62E-05 | 0.04 | S100A12 | S100 calcium binding protein A12 |
| 84984 | 5.2098 | 5.76E-05 | 0.04 | CEP19 | centrosomal protein 19 |
| 147991 | 5.2229 | 0.00010934 | 0.06 | DPY19L3 | dpy-19 like C-mannosyltransferase 3 |
| 27063 | 5.2237 | 0.00038505 | 0.12 | ANKRD1 | ankyrin repeat domain 1 |
| 4318 | 5.2402 | 2.30E-05 | 0.02 | MMP9 | matrix metallopeptidase 9 |
| 79989 | 5.2975 | 2.71E-05 | 0.02 | TTC26 | tetratricopeptide repeat domain 26 |
| 23569 | 5.3678 | 0.00012088 | 0.07 | PADI4 | peptidyl arginine deiminase 4 |
| 8653 | 5.517 | 0.0095227 | 0.36 | DDX3Y | DEAD-box helicase 3 Y-linked |
| 4286 | 5.603 | 1.43E-06 | 0.01 | MITF | melanocyte inducing transcription factor |
| 64386 | 5.6302 | 0.00016164 | 0.07 | MMP25 | matrix metallopeptidase 25 |
| 63926 | 5.678 | 2.10E-06 | 0.01 | ANKEF1 | ankyrin repeat and EF-hand domain containing 1 |
| 25984 | 5.8028 | 2.45E-05 | 0.02 | KRT23 | keratin 23 |
| 353511 | 6.0439 | 6.16E-07 | 0.00 | PKD1P6 | "polycystin 1, transient receptor potential channel interacting pseudogene 6" |
| 249 | 6.149 | 4.10E-06 | 0.01 | ALPL | "alkaline phosphatase, biomineralization associated" |
| 79689 | 6.2843 | 8.06E-07 | 0.00 | STEAP4 | STEAP4 metalloreductase |

logFC: log Fold Change; adj.p.val: adjusted p value
