# Supplementary material for: Association of Circulating, Inflammatory-Response Exosomal mRNAs With Acute Myocardial Infarction
Source: Front Cardiovasc Med. 2021 Aug 19;8:712061. doi: 10.3389/fcvm.2021.712061 (PMC8418229; doi:10.3389/fcvm.2021.712061)
Supplement: Supplementary file 2 [file Table_2.DOCX]

Table S2 Functional profiles (Gene Ontology) of the different exosomal mRNAs in AMI group compared with the control group

| ONTOLOGY | ID | Description | Gene Ratio | p value | adjusted p value | q value | geneID |
| --- | --- | --- | --- | --- | --- | --- | --- |
| BP | GO:0043312 | neutrophil degranulation | 24/242 | 2.17E-08 | 2.87E-05 | 2.76E-05 | MMP9/S100A12/HP/MMP25/S100A8/PYGL/PGLYRP1/FPR1/PADI2/PTAFR/S100A9/CXCR2/CRISPLD2/VNN1/RNASET2/FCAR/MNDA/ELANE/CD14/SDCBP/MPO/ANO6/VCL/CDA |
| BP | GO:0002283 | neutrophil activation involved in immune response | 24/242 | 2.44E-08 | 2.87E-05 | 2.76E-05 | MMP9/S100A12/HP/MMP25/S100A8/PYGL/PGLYRP1/FPR1/PADI2/PTAFR/S100A9/CXCR2/CRISPLD2/VNN1/RNASET2/FCAR/MNDA/ELANE/CD14/SDCBP/MPO/ANO6/VCL/CDA |
| BP | GO:0042119 | neutrophil activation | 24/242 | 3.58E-08 | 2.87E-05 | 2.76E-05 | MMP9/S100A12/HP/MMP25/S100A8/PYGL/PGLYRP1/FPR1/PADI2/PTAFR/S100A9/CXCR2/CRISPLD2/VNN1/RNASET2/FCAR/MNDA/ELANE/CD14/SDCBP/MPO/ANO6/VCL/CDA |
| BP | GO:0002446 | neutrophil mediated immunity | 24/242 | 3.72E-08 | 2.87E-05 | 2.76E-05 | MMP9/S100A12/HP/MMP25/S100A8/PYGL/PGLYRP1/FPR1/PADI2/PTAFR/S100A9/CXCR2/CRISPLD2/VNN1/RNASET2/FCAR/MNDA/ELANE/CD14/SDCBP/MPO/ANO6/VCL/CDA |
| BP | GO:0050832 | defense response to fungus | 6/242 | 1.18E-05 | 0.007304 | 0.007031 | S100A12/S100A8/S100A9/ELANE/MPO/DEFA3 |
| BP | GO:0002523 | leukocyte migration involved in inflammatory response | 4/242 | 2.49E-05 | 0.012805 | 0.012326 | SLAMF8/S100A8/S100A9/ELANE |
| BP | GO:0019730 | antimicrobial humoral response | 9/242 | 3.04E-05 | 0.013425 | 0.012923 | FGA/S100A12/S100A8/PGLYRP1/S100A9/ELANE/DEFA3/CXCL5/FGB |
| BP | GO:0034116 | positive regulation of heterotypic cell-cell adhesion | 4/242 | 4.43E-05 | 0.01526 | 0.014689 | FGA/FGG/ALOX15/FGB |
| BP | GO:0002576 | platelet degranulation | 9/242 | 4.45E-05 | 0.01526 | 0.014689 | FGA/TF/FGG/SCCPDH/ITGA2B/VEGFA/MMRN1/VCL/FGB |
| BP | GO:0009620 | response to fungus | 6/242 | 5.51E-05 | 0.017012 | 0.016376 | S100A12/S100A8/S100A9/ELANE/MPO/DEFA3 |
| BP | GO:0010038 | response to metal ion | 15/242 | 8.62E-05 | 0.022956 | 0.022098 | FGA/TF/MMP9/FGG/S100A8/BMP6/CD14/TNNT2/ALOX15/IGFBP2/NQO1/UROS/NEDD4L/FGB/XRCC4 |
| BP | GO:0003018 | vascular process in circulatory system | 10/242 | 8.92E-05 | 0.022956 | 0.022098 | FGA/FGG/PTAFR/CRP/CXCR2/SOD2/BMP6/VEGFA/FGB/RGS2 |
| BP | GO:0042742 | defense response to bacterium | 14/242 | 0.000109 | 0.025956 | 0.024986 | FGA/S100A12/SLAMF8/HP/S100A8/PGLYRP1/CRP/S100A9/ADAMTS5/ELANE/MPO/DEFA3/TLR5/FGB |
| BP | GO:0030595 | leukocyte chemotaxis | 11/242 | 0.000171 | 0.037674 | 0.036265 | S100A12/SLAMF8/S100A8/PADI2/S100A9/CXCR2/DAPK2/TNFSF14/ANO6/CXCL5/VEGFA |
| CC | GO:0034774 | secretory granule lumen | 23/249 | 2.14E-11 | 7.93E-09 | 7.58E-09 | FGA/TF/FGG/S100A12/HP/S100A8/PYGL/PGLYRP1/PADI2/S100A9/CRISPLD2/RNASET2/MNDA/ELANE/SDCBP/SCCPDH/MPO/DEFA3/VEGFA/MMRN1/VCL/FGB/CDA |
| CC | GO:0060205 | cytoplasmic vesicle lumen | 23/249 | 6.06E-11 | 7.93E-09 | 7.58E-09 | FGA/TF/FGG/S100A12/HP/S100A8/PYGL/PGLYRP1/PADI2/S100A9/CRISPLD2/RNASET2/MNDA/ELANE/SDCBP/SCCPDH/MPO/DEFA3/VEGFA/MMRN1/VCL/FGB/CDA |
| CC | GO:0031983 | vesicle lumen | 23/249 | 6.43E-11 | 7.93E-09 | 7.58E-09 | FGA/TF/FGG/S100A12/HP/S100A8/PYGL/PGLYRP1/PADI2/S100A9/CRISPLD2/RNASET2/MNDA/ELANE/SDCBP/SCCPDH/MPO/DEFA3/VEGFA/MMRN1/VCL/FGB/CDA |
| CC | GO:0072562 | blood microparticle | 9/249 | 0.000107 | 0.007416 | 0.007089 | FGA/TF/FGG/HP/CIB2/SDCBP/APOA2/ITGA2B/FGB |
| CC | GO:0031091 | platelet alpha granule | 7/249 | 0.000155 | 0.007416 | 0.007089 | FGA/FGG/SCCPDH/ITGA2B/VEGFA/MMRN1/FGB |
| CC | GO:0035578 | azurophil granule lumen | 7/249 | 0.000155 | 0.007416 | 0.007089 | PADI2/RNASET2/MNDA/ELANE/SDCBP/MPO/DEFA3 |
| CC | GO:0005766 | primary lysosome | 9/249 | 0.00016 | 0.007416 | 0.007089 | FPR1/PADI2/VNN1/RNASET2/MNDA/ELANE/SDCBP/MPO/DEFA3 |
| CC | GO:0042582 | azurophil granule | 9/249 | 0.00016 | 0.007416 | 0.007089 | FPR1/PADI2/VNN1/RNASET2/MNDA/ELANE/SDCBP/MPO/DEFA3 |
| CC | GO:0031093 | platelet alpha granule lumen | 6/249 | 0.0002 | 0.008229 | 0.007866 | FGA/FGG/SCCPDH/VEGFA/MMRN1/FGB |
| CC | GO:0005775 | vacuolar lumen | 9/249 | 0.000348 | 0.012885 | 0.012317 | PADI2/RNASET2/DAPK2/MNDA/ELANE/TCN2/SDCBP/MPO/DEFA3 |
| CC | GO:0070820 | tertiary granule | 8/249 | 0.001164 | 0.03914 | 0.037414 | MMP9/HP/PGLYRP1/FPR1/PTAFR/FCAR/ANO6/CDA |
| CC | GO:0030667 | secretory granule membrane | 11/249 | 0.001494 | 0.046079 | 0.044047 | MMP25/FPR1/PTAFR/CXCR2/ACRBP/VNN1/FCAR/CD14/ANO6/ITGA2B/CCDC136 |
| MF | GO:0050786 | RAGE receptor binding | 4/242 | 1.04E-05 | 0.005 | 0.004758 | S100A12/S100A8/FPR1/S100A9 |

BP: biological processes; CC: cellular components; MF: molecular functions
