# Supplementary material for: Association of Circulating, Inflammatory-Response Exosomal mRNAs With Acute Myocardial Infarction
Source: Front Cardiovasc Med. 2021 Aug 19;8:712061. doi: 10.3389/fcvm.2021.712061 (PMC8418229; doi:10.3389/fcvm.2021.712061)
Supplement: Supplementary file 3 [file Table_3.DOCX]

Table S3 The different exosomal mRNAs in AMI group compared with the CAD group

| EntrezID | logFC | p.value | adj.p.val | symbols | name |
| --- | --- | --- | --- | --- | --- |
| 106480134 | 3.67 | 4.46E-05 | 0.082858 | RNU6-1300P | "RNA, U6 small nuclear 1300, pseudogene" |
| 6635 | 3.46 | 0.000174 | 0.17969 | SNRPE | small nuclear ribonucleoprotein polypeptide E |
| 6283 | 3.13 | 0.000273 | 0.17969 | S100A12 | S100 calcium binding protein A12 |
| 6288 | 2.72 | 0.009061 | 0.44147 | SAA1 | serum amyloid A1 |
| 81034 | 2.52 | 0.000266 | 0.17969 | SLC25A32 | solute carrier family 25 member 32 |
| 51188 | 2.47 | 0.002499 | 0.36705 | SS18L2 | SS18 like 2 |
| 84292 | 2.46 | 0.000828 | 0.25676 | WDR83 | WD repeat domain 83 |
| 152195 | 2.45 | 0.000298 | 0.17969 | NUDT16P1 | nudix hydrolase 16 pseudogene 1 |
| 5004 | 2.44 | 0.007564 | 0.43873 | ORM1 | orosomucoid 1 |
| 51023 | 2.39 | 0.001412 | 0.32819 | MRPS18C | mitochondrial ribosomal protein S18C |
| 1475 | 2.39 | 0.001988 | 0.33114 | CSTA | cystatin A |
| 9021 | 2.37 | 0.000536 | 0.23545 | SOCS3 | suppressor of cytokine signaling 3 |
| 348093 | 2.36 | 0.005517 | 0.42514 | RBPMS2 | "RNA binding protein, mRNA processing factor 2" |
| 388753 | 2.35 | 0.006376 | 0.42514 | COA6 | cytochrome c oxidase assembly factor 6 |
| 249 | 2.31 | 0.000415 | 0.2078 | ALPL | "alkaline phosphatase, biomineralization associated" |
| 401303 | 2.27 | 0.003451 | 0.37583 | ZNF815P | "zinc finger protein 815, pseudogene" |
| 84519 | 2.25 | 0.002086 | 0.33791 | ACRBP | acrosin binding protein |
| 140885 | 2.17 | 0.000743 | 0.24982 | SIRPA | signal regulatory protein alpha |
| 10911 | 2.15 | 0.006965 | 0.42578 | UTS2 | urotensin 2 |
| 93973 | 2.15 | 0.000612 | 0.23545 | ACTR8 | actin related protein 8 |
| 3557 | 2.14 | 0.001094 | 0.2808 | IL1RN | interleukin 1 receptor antagonist |
| 27180 | 2.12 | 0.00183 | 0.33114 | SIGLEC9 | sialic acid binding Ig like lectin 9 |
| 6231 | 2.11 | 0.007008 | 0.42578 | RPS26 | ribosomal protein S26 |
| 7276 | 2.06 | 0.008032 | 0.44011 | TTR | transthyretin |
| 710 | 2.05 | 0.00196 | 0.33114 | SERPING1 | serpin family G member 1 |
| 55003 | 2.04 | 0.003017 | 0.36926 | PAK1IP1 | PAK1 interacting protein 1 |
| 284029 | 2.04 | 0.005083 | 0.40527 | LINC00324 | long intergenic non-protein coding RNA 324 |
| 60675 | 2.02 | 0.006218 | 0.42514 | PROK2 | prokineticin 2 |
| 27075 | 2.00 | 0.004049 | 0.39224 | TSPAN13 | tetraspanin 13 |
| 11157 | 2.00 | 0.002735 | 0.36805 | LSM6 | "LSM6 homolog, U6 small nuclear RNA and mRNA degradation associated" |
| 8624 | 1.97 | 0.002574 | 0.36761 | PSMG1 | proteasome assembly chaperone 1 |
| 9064 | 1.96 | 0.001712 | 0.33114 | MAP3K6 | mitogen-activated protein kinase kinase kinase 6 |
| 9694 | 1.96 | 0.002929 | 0.36926 | EMC2 | ER membrane protein complex subunit 2 |
| 51562 | 1.96 | 0.007706 | 0.43873 | MBIP | MAP3K12 binding inhibitory protein 1 |
| 55617 | 1.94 | 0.004065 | 0.39224 | TASP1 | taspase 1 |
| 170622 | 1.93 | 0.006239 | 0.42514 | COMMD6 | COMM domain containing 6 |
| 6279 | 1.91 | 9.37E-05 | 0.13066 | S100A8 | S100 calcium binding protein A8 |
| 440145 | 1.89 | 0.005157 | 0.41708 | MZT1 | mitotic spindle organizing protein 1 |
| 366 | 1.89 | 0.007427 | 0.43873 | AQP9 | aquaporin 9 |
| 4318 | 1.88 | 0.003564 | 0.37583 | MMP9 | matrix metallopeptidase 9 |
| 23569 | 1.88 | 0.001089 | 0.2808 | PADI4 | peptidyl arginine deiminase 4 |
| 90326 | 1.88 | 0.006613 | 0.42514 | THAP3 | THAP domain containing 3 |
| 441386 | 1.88 | 0.005268 | 0.4188 | RPS26P3 | ribosomal protein S26 pseudogene 3 |
| 6948 | 1.87 | 0.006691 | 0.42514 | TCN2 | transcobalamin 2 |
| 58484 | 1.85 | 0.000705 | 0.24982 | NLRC4 | NLR family CARD domain containing 4 |
| 5165 | 1.82 | 0.002334 | 0.3669 | PDK3 | pyruvate dehydrogenase kinase 3 |
| 1401 | 1.82 | 0.006353 | 0.42514 | CRP | C-reactive protein |
| 112770 | 1.82 | 0.003285 | 0.36926 | GLMP | glycosylated lysosomal membrane protein |
| 8649 | 1.82 | 0.002738 | 0.36805 | LAMTOR3 | "late endosomal/lysosomal adaptor, MAPK and MTOR activator 3" |
| 10135 | 1.81 | 0.000199 | 0.17969 | NAMPT | nicotinamide phosphoribosyltransferase |
| 58527 | 1.81 | 0.000269 | 0.17969 | ABRACL | ABRA C-terminal like |
| 55332 | 1.79 | 0.002331 | 0.3669 | DRAM1 | DNA damage regulated autophagy modulator 1 |
| 646309 | 1.78 | 0.000142 | 0.17611 | NAMPTP1 | nicotinamide phosphoribosyltransferase pseudogene 1 |
| 7518 | 1.78 | 0.005124 | 0.40658 | XRCC4 | X-ray repair cross complementing 4 |
| 2266 | 1.77 | 0.001946 | 0.33114 | FGG | fibrinogen gamma chain |
| 23483 | 1.77 | 0.001503 | 0.32949 | TGDS | "TDP-glucose 4,6-dehydratase" |
| 100113407 | 1.76 | 0.000799 | 0.25461 | TMEM170B | transmembrane protein 170B |
| 3416 | 1.76 | 0.001071 | 0.2808 | IDE | insulin degrading enzyme |
| 7739 | 1.75 | 0.004366 | 0.39733 | ZNF185 | zinc finger protein 185 with LIM domain |
| 79369 | 1.73 | 0.007 | 0.42578 | B3GNT4 | "UDP-GlcNAc:betaGal beta-1,3-N-acetylglucosaminyltransferase 4" |
| 5836 | 1.72 | 0.000302 | 0.17969 | PYGL | glycogen phosphorylase L |
| 10552 | 1.71 | 0.003874 | 0.39224 | ARPC1A | actin related protein 2/3 complex subunit 1A |
| 87178 | 1.70 | 0.007804 | 0.44011 | PNPT1 | polyribonucleotide nucleotidyltransferase 1 |
| 84340 | 1.67 | 0.004285 | 0.39733 | GFM2 | G elongation factor mitochondrial 2 |
| 64225 | 1.65 | 0.001819 | 0.33114 | ATL2 | atlastin GTPase 2 |
| 4311 | 1.61 | 0.005883 | 0.42514 | MME | membrane metalloendopeptidase |
| 51170 | 1.60 | 0.008766 | 0.44107 | HSD17B11 | hydroxysteroid 17-beta dehydrogenase 11 |
| 10550 | 1.58 | 0.001593 | 0.33114 | ARL6IP5 | ADP ribosylation factor like GTPase 6 interacting protein 5 |
| 55858 | 1.58 | 0.00909 | 0.44525 | TMEM165 | transmembrane protein 165 |
| 8611 | 1.57 | 0.008372 | 0.44011 | PLPP1 | phospholipid phosphatase 1 |
| 79989 | 1.57 | 0.004486 | 0.40277 | TTC26 | tetratricopeptide repeat domain 26 |
| 91801 | 1.57 | 0.00184 | 0.33114 | ALKBH8 | "alkB homolog 8, tRNA methyltransferase" |
| 7099 | 1.57 | 2.76E-05 | 0.061593 | TLR4 | toll like receptor 4 |
| 7006 | 1.56 | 0.002723 | 0.36805 | TEC | tec protein tyrosine kinase |
| 55793 | 1.54 | 0.00656 | 0.42514 | MINDY1 | MINDY lysine 48 deubiquitinase 1 |
| 25801 | 1.53 | 0.00243 | 0.36705 | GCA | grancalcin |
| 353511 | 1.53 | 0.006913 | 0.42514 | PKD1P6 | "polycystin 1, transient receptor potential channel interacting pseudogene 6" |
| 4212 | 1.52 | 0.004636 | 0.40277 | MEIS2 | Meis homeobox 2 |
| 4332 | 1.48 | 0.000592 | 0.23545 | MNDA | myeloid cell nuclear differentiation antigen |
| 7321 | 1.48 | 0.007274 | 0.43873 | UBE2D1 | ubiquitin conjugating enzyme E2 D1 |
| 25987 | 1.47 | 0.006863 | 0.42514 | TSKU | "tsukushi, small leucine rich proteoglycan" |
| 2212 | 1.46 | 0.001107 | 0.2808 | FCGR2A | Fc fragment of IgG receptor IIa |
| 9586 | 1.44 | 0.003015 | 0.36926 | CREB5 | cAMP responsive element binding protein 5 |
| 81671 | 1.44 | 0.008858 | 0.44147 | VMP1 | vacuole membrane protein 1 |
| 3772 | 1.42 | 0.008556 | 0.44011 | KCNJ15 | potassium inwardly rectifying channel subfamily J member 15 |
| 29940 | 1.42 | 0.004292 | 0.39733 | DSE | dermatan sulfate epimerase |
| 91661 | 1.41 | 0.001506 | 0.32949 | ZNF765 | zinc finger protein 765 |
| 23306 | 1.40 | 0.006943 | 0.42578 | NEMP1 | nuclear envelope integral membrane protein 1 |
| 51524 | 1.40 | 0.00835 | 0.44011 | TMEM138 | transmembrane protein 138 |
| 2180 | 1.39 | 0.004452 | 0.40277 | ACSL1 | acyl-CoA synthetase long chain family member 1 |
| 401494 | 1.39 | 0.004814 | 0.40527 | HACD4 | 3-hydroxyacyl-CoA dehydratase 4 |
| 29085 | 1.38 | 0.006372 | 0.42514 | PHPT1 | phosphohistidine phosphatase 1 |
| 9848 | 1.38 | 0.005283 | 0.42514 | MFAP3L | microfibril associated protein 3 like |
| 120892 | 1.35 | 0.0056 | 0.42514 | LRRK2 | leucine rich repeat kinase 2 |
| 5238 | 1.34 | 0.007247 | 0.43873 | PGM3 | phosphoglucomutase 3 |
| 116068 | 1.34 | 0.004794 | 0.40527 | LYSMD3 | LysM domain containing 3 |
| 5525 | 1.32 | 0.000428 | 0.2078 | PPP2R5A | protein phosphatase 2 regulatory subunit B'alpha |
| 84243 | 1.32 | 0.007881 | 0.44011 | ZDHHC18 | zinc finger DHHC-type palmitoyltransferase 18 |
| 1843 | 1.31 | 0.004334 | 0.39733 | DUSP1 | dual specificity phosphatase 1 |
| 6280 | 1.31 | 0.000873 | 0.25701 | S100A9 | S100 calcium binding protein A9 |
| 22936 | 1.28 | 0.006923 | 0.42514 | ELL2 | elongation factor for RNA polymerase II 2 |
| 11275 | 1.25 | 0.00506 | 0.40527 | KLHL2 | kelch like family member 2 |
| 79660 | 1.23 | 0.007028 | 0.43165 | PPP1R3B | protein phosphatase 1 regulatory subunit 3B |
| 90459 | 1.23 | 0.008939 | 0.44147 | ERI1 | exoribonuclease 1 |
| 134492 | 1.22 | 0.007238 | 0.43871 | NUDCD2 | NudC domain containing 2 |
| 10342 | 1.18 | 0.003833 | 0.39224 | TFG | trafficking from ER to golgi regulator |
| 4084 | 1.17 | 0.002824 | 0.36926 | MXD1 | MAX dimerization protein 1 |
| 1955 | 1.14 | 0.004404 | 0.39733 | MEGF9 | multiple EGF like domains 9 |
| 80063 | 1.14 | 0.005887 | 0.42514 | ATF7IP2 | activating transcription factor 7 interacting protein 2 |
| 6282 | 1.14 | 0.002458 | 0.36705 | S100A11 | S100 calcium binding protein A11 |
| 401152 | 1.14 | 0.006796 | 0.42514 | C4orf3 | chromosome 4 open reading frame 3 |
| 55327 | 1.11 | 0.005139 | 0.40658 | LIN7C | "lin-7 homolog C, crumbs cell polarity complex component" |
| 5817 | 1.10 | 0.008772 | 0.44107 | PVR | PVR cell adhesion molecule |
| 5884 | 1.08 | 0.007048 | 0.43509 | RAD17 | RAD17 checkpoint clamp loader component |
| 6648 | 1.08 | 0.003416 | 0.37385 | SOD2 | superoxide dismutase 2 |
| 11213 | 1.07 | 0.003706 | 0.38534 | IRAK3 | interleukin 1 receptor associated kinase 3 |
| 11237 | 1.06 | 0.00887 | 0.44147 | RNF24 | ring finger protein 24 |
| 6386 | 1.04 | 0.006931 | 0.42514 | SDCBP | syndecan binding protein |
| 23341 | 1.04 | 0.008604 | 0.44107 | DNAJC16 | DnaJ heat shock protein family (Hsp40) member C16 |
| 64960 | 1.04 | 0.008009 | 0.44011 | MRPS15 | mitochondrial ribosomal protein S15 |
| 79735 | -1.05 | 0.006717 | 0.42514 | TBC1D17 | TBC1 domain family member 17 |
| 11142 | -1.05 | 0.006085 | 0.42514 | PKIG | cAMP-dependent protein kinase inhibitor gamma |
| 3633 | -1.08 | 0.006118 | 0.42514 | INPP5B | inositol polyphosphate-5-phosphatase B |
| 7554 | -1.08 | 0.007755 | 0.43873 | ZNF8 | zinc finger protein 8 |
| 285025 | -1.13 | 0.008672 | 0.44107 | CCDC141 | coiled-coil domain containing 141 |
| 7220 | -1.13 | 0.006849 | 0.42514 | TRPC1 | transient receptor potential cation channel subfamily C member 1 |
| 5426 | -1.13 | 0.00315 | 0.36926 | POLE | "DNA polymerase epsilon, catalytic subunit" |
| 23666 | -1.14 | 0.006006 | 0.42514 | UBBP4 | ubiquitin B pseudogene 4 |
| 653784 | -1.18 | 0.00412 | 0.39733 | MZT2A | mitotic spindle organizing protein 2A |
| 10016 | -1.20 | 0.001589 | 0.33114 | PDCD6 | programmed cell death 6 |
| 29062 | -1.22 | 0.008649 | 0.44107 | WDR91 | WD repeat domain 91 |
| 642741 | -1.22 | 0.008531 | 0.44011 | RPL3P7 | ribosomal protein L3 pseudogene 7 |
| 162466 | -1.23 | 0.007279 | 0.43873 | PHOSPHO1 | phosphoethanolamine/phosphocholine phosphatase 1 |
| 81931 | -1.26 | 0.009166 | 0.44939 | ZNF93 | zinc finger protein 93 |
| 100270840 | -1.26 | 0.006531 | 0.42514 | RPL5P23 | ribosomal protein L5 pseudogene 23 |
| 149013 | -1.27 | 0.000903 | 0.25829 | NBPF12 | NBPF member 12 |
| 8406 | -1.29 | 0.008701 | 0.44107 | SRPX | sushi repeat containing protein X-linked |
| 26153 | -1.30 | 0.004558 | 0.40277 | KIF26A | kinesin family member 26A |
| 29115 | -1.32 | 0.000875 | 0.25701 | SAP30BP | SAP30 binding protein |
| 100873463 | -1.33 | 0.003113 | 0.36926 | RNA5SP202 | "RNA, 5S ribosomal pseudogene 202" |
| 100270839 | -1.33 | 0.004429 | 0.40045 | RPL5P17 | ribosomal protein L5 pseudogene 17 |
| 29933 | -1.34 | 0.006606 | 0.42514 | GPR132 | G protein-coupled receptor 132 |
| 84836 | -1.34 | 0.007958 | 0.44011 | ABHD14B | abhydrolase domain containing 14B |
| 7058 | -1.35 | 0.000579 | 0.23545 | THBS2 | thrombospondin 2 |
| 166336 | -1.37 | 0.004398 | 0.39733 | PRICKLE2 | prickle planar cell polarity protein 2 |
| 100505495 | -1.37 | 0.007954 | 0.44011 | PCAT19 | prostate cancer associated transcript 19 |
| 8904 | -1.38 | 0.008874 | 0.44147 | CPNE1 | copine 1 |
| 100507458 | -1.39 | 0.004961 | 0.40527 | ZNF213-AS1 | ZNF213 antisense RNA 1 (head to head) |
| 56996 | -1.39 | 0.006254 | 0.42514 | SLC12A9 | solute carrier family 12 member 9 |
| 126208 | -1.42 | 0.009073 | 0.44471 | ZNF787 | zinc finger protein 787 |
| 64949 | -1.42 | 0.003309 | 0.37034 | MRPS26 | mitochondrial ribosomal protein S26 |
| 5261 | -1.45 | 0.007177 | 0.43783 | PHKG2 | phosphorylase kinase catalytic subunit gamma 2 |
| 338657 | -1.45 | 0.006881 | 0.42514 | CCDC84 | coiled-coil domain containing 84 |
| 79865 | -1.50 | 0.003352 | 0.37367 | TREML2 | triggering receptor expressed on myeloid cells like 2 |
| 54758 | -1.50 | 0.002603 | 0.36761 | KLHDC4 | kelch domain containing 4 |
| 146857 | -1.50 | 0.002089 | 0.33791 | SLFN13 | schlafen family member 13 |
| 231 | -1.53 | 0.005097 | 0.40527 | AKR1B1 | aldo-keto reductase family 1 member B |
| 1628 | -1.55 | 0.003219 | 0.36926 | DBP | D-box binding PAR bZIP transcription factor |
| 26094 | -1.56 | 0.005457 | 0.42514 | DCAF4 | DDB1 and CUL4 associated factor 4 |
| 6423 | -1.56 | 0.00808 | 0.44011 | SFRP2 | secreted frizzled related protein 2 |
| 9020 | -1.56 | 0.001882 | 0.33114 | MAP3K14 | mitogen-activated protein kinase kinase kinase 14 |
| 253039 | -1.60 | 0.002718 | 0.36805 | CUTALP | "cutA divalent cation tolerance homolog-like, pseudogene" |
| 80317 | -1.60 | 0.008804 | 0.44107 | ZKSCAN3 | zinc finger with KRAB and SCAN domains 3 |
| 79600 | -1.63 | 0.008577 | 0.44011 | TCTN1 | tectonic family member 1 |
| 1292 | -1.63 | 0.008139 | 0.44011 | COL6A2 | collagen type VI alpha 2 chain |
| 730029 | -1.65 | 0.004071 | 0.39733 | RPSAP19 | ribosomal protein SA pseudogene 19 |
| 1716 | -1.66 | 0.008101 | 0.44011 | DGUOK | deoxyguanosine kinase |
| 84514 | -1.67 | 0.007043 | 0.43328 | GHDC | GH3 domain containing |
| 11132 | -1.68 | 0.000954 | 0.26605 | CAPN10 | calpain 10 |
| 683 | -1.69 | 0.003033 | 0.36926 | BST1 | bone marrow stromal cell antigen 1 |
| 55257 | -1.69 | 0.007855 | 0.44011 | MRGBP | MRG domain binding protein |
| 326279 | -1.70 | 0.001799 | 0.33114 | RPL15P2 | ribosomal protein L15 pseudogene 2 |
| 55308 | -1.72 | 0.005283 | 0.42226 | DDX19A | DEAD-box helicase 19A |
| 23324 | -1.72 | 0.00139 | 0.32819 | MAN2B2 | mannosidase alpha class 2B member 2 |
| 9880 | -1.72 | 0.00354 | 0.37583 | ZBTB39 | zinc finger and BTB domain containing 39 |
| 8412 | -1.73 | 0.008752 | 0.44107 | BCAR3 | "BCAR3 adaptor protein, NSP family member" |
| 10847 | -1.74 | 2.10E-05 | 0.05855 | SRCAP | Snf2 related CREBBP activator protein |
| 3913 | -1.75 | 6.79E-05 | 0.1083 | LAMB2 | laminin subunit beta 2 |
| 3039 | -1.76 | 0.001256 | 0.30473 | HBA1 | hemoglobin subunit alpha 1 |
| 5761 | -1.81 | 0.003192 | 0.36926 | PTMAP4 | prothymosin alpha pseudogene 4 |
| 1917 | -1.81 | 0.001783 | 0.33114 | EEF1A2 | eukaryotic translation elongation factor 1 alpha 2 |
| 9214 | -1.81 | 0.007651 | 0.43873 | FCMR | Fc fragment of IgM receptor |
| 252969 | -1.81 | 0.006702 | 0.42514 | NEIL2 | nei like DNA glycosylase 2 |
| 933 | -1.82 | 0.007288 | 0.43873 | CD22 | CD22 molecule |
| 147011 | -1.82 | 0.00033 | 0.17969 | PROCA1 | protein interacting with cyclin A1 |
| 7106 | -1.84 | 0.002946 | 0.36926 | TSPAN4 | tetraspanin 4 |
| 79175 | -1.84 | 0.003656 | 0.37998 | ZNF343 | zinc finger protein 343 |
| 79639 | -1.84 | 0.003746 | 0.38601 | TMEM53 | transmembrane protein 53 |
| 100133991 | -1.85 | 0.001852 | 0.33114 | MAP3K14-AS1 | MAP3K14 antisense RNA 1 |
| 112752 | -1.85 | 0.006793 | 0.42514 | IFT43 | intraflagellar transport 43 |
| 9925 | -1.86 | 0.003248 | 0.36926 | ZBTB5 | zinc finger and BTB domain containing 5 |
| 10067 | -1.87 | 0.005125 | 0.40658 | SCAMP3 | secretory carrier membrane protein 3 |
| 81571 | -1.90 | 0.001923 | 0.33114 | MIR600HG | MIR600 host gene |
| 23070 | -1.91 | 0.002587 | 0.36761 | CMTR1 | cap methyltransferase 1 |
| 9546 | -1.92 | 0.001888 | 0.33114 | APBA3 | amyloid beta precursor protein binding family A member 3 |
| 222229 | -1.92 | 0.001224 | 0.30359 | LRWD1 | leucine rich repeats and WD repeat domain containing 1 |
| 115650 | -1.93 | 0.0025 | 0.36705 | TNFRSF13C | TNF receptor superfamily member 13C |
| 2672 | -1.93 | 0.007002 | 0.42578 | GFI1 | growth factor independent 1 transcriptional repressor |
| 138428 | -1.95 | 0.000338 | 0.17969 | PTRH1 | peptidyl-tRNA hydrolase 1 homolog |
| 147968 | -1.98 | 0.007494 | 0.43873 | CAPN12 | calpain 12 |
| 56271 | -2.03 | 0.005758 | 0.42514 | BEX4 | brain expressed X-linked 4 |
| 84277 | -2.04 | 0.001927 | 0.33114 | DNAJC30 | DnaJ heat shock protein family (Hsp40) member C30 |
| 971 | -2.05 | 0.003079 | 0.36926 | CD72 | CD72 molecule |
| 5079 | -2.06 | 3.95E-06 | 0.022043 | PAX5 | paired box 5 |
| 64167 | -2.08 | 0.000221 | 0.17969 | ERAP2 | endoplasmic reticulum aminopeptidase 2 |
| 3495 | -2.09 | 0.004077 | 0.39733 | IGHD | immunoglobulin heavy constant delta |
| 84975 | -2.11 | 0.002372 | 0.36705 | MFSD5 | major facilitator superfamily domain containing 5 |
| 2805 | -2.11 | 0.004837 | 0.40527 | GOT1 | glutamic-oxaloacetic transaminase 1 |
| 200035 | -2.12 | 0.003215 | 0.36926 | NUDT17 | nudix hydrolase 17 |
| 80194 | -2.13 | 0.000761 | 0.24982 | TMEM134 | transmembrane protein 134 |
| 151887 | -2.13 | 0.00022 | 0.17969 | CCDC80 | coiled-coil domain containing 80 |
| 84727 | -2.20 | 0.006597 | 0.42514 | SPSB2 | splA/ryanodine receptor domain and SOCS box containing 2 |
| 404550 | -2.27 | 0.004735 | 0.40277 | C16orf74 | chromosome 16 open reading frame 74 |
| 2202 | -2.31 | 0.001505 | 0.32949 | EFEMP1 | EGF containing fibulin extracellular matrix protein 1 |
| 26866 | -2.36 | 0.004719 | 0.40277 | RNU1-28P | "RNA, U1 small nuclear 28, pseudogene" |
| 152789 | -2.37 | 0.003609 | 0.37941 | JAKMIP1 | janus kinase and microtubule interacting protein 1 |
| 1840 | -2.41 | 0.000722 | 0.24982 | DTX1 | deltex E3 ubiquitin ligase 1 |
| 4049 | -2.47 | 0.004784 | 0.40527 | LTA | lymphotoxin alpha |
| 84619 | -2.48 | 0.000675 | 0.24982 | ZGPAT | zinc finger CCCH-type and G-patch domain containing |
| 5806 | -2.50 | 0.00357 | 0.37772 | PTX3 | pentraxin 3 |
| 100169760 | -2.50 | 0.002906 | 0.36926 | RNA5S9 | "RNA, 5S ribosomal 9" |
| 25776 | -2.53 | 0.005766 | 0.42514 | CBY1 | "chibby family member 1, beta catenin antagonist" |
| 10126 | -2.57 | 0.00308 | 0.36926 | DNAL4 | dynein axonemal light chain 4 |
| 54859 | -2.58 | 0.000567 | 0.23545 | ELP6 | elongator acetyltransferase complex subunit 6 |
| 8115 | -2.76 | 0.000331 | 0.17969 | TCL1A | T cell leukemia/lymphoma 1A |
| 10518 | -2.95 | 1.01E-05 | 0.037459 | CIB2 | calcium and integrin binding family member 2 |
| 124512 | -3.02 | 0.000502 | 0.23327 | METTL23 | methyltransferase like 23 |
| 28950 | -3.28 | 0.008658 | 0.44107 | IGKJ1 | immunoglobulin kappa joining 1 |
| 2335 | -3.33 | 4.21E-07 | 0.0046926 | FN1 | fibronectin 1 |
| 28522 | -3.77 | 0.008841 | 0.44107 | TRDJ1 | T cell receptor delta joining 1 |

logFC: log Fold Change; adj.p.val: adjusted p value
