# Supplementary material for: Association of Circulating, Inflammatory-Response Exosomal mRNAs With Acute Myocardial Infarction
Source: Front Cardiovasc Med. 2021 Aug 19;8:712061. doi: 10.3389/fcvm.2021.712061 (PMC8418229; doi:10.3389/fcvm.2021.712061)
Supplement: Supplementary file 4 [file Table_4.DOCX]

Table S4 Functional profiles (Gene Ontology) of the different exosomal mRNAs in AMI group compared with the CAD group

| ONTOLOGY | ID | Description | Gene Ratio | p value | adjusted p value | q value | geneID |
| --- | --- | --- | --- | --- | --- | --- | --- |
| BP | GO:0043312 | neutrophil degranulation | 20/196 | 2.05E-07 | 0.000214 | 0.000203 | S100A8/S100A12/PYGL/MNDA/SIRPA/S100A9/FCGR2A/SIGLEC9/GCA/S100A11/LAMTOR3/BST1/MMP9/PTX3/MME/SDCBP/GHDC/ORM1/TTR/CPNE1 |
| BP | GO:0002283 | neutrophil activation involved in immune response | 20/196 | 2.26E-07 | 0.000214 | 0.000203 | S100A8/S100A12/PYGL/MNDA/SIRPA/S100A9/FCGR2A/SIGLEC9/GCA/S100A11/LAMTOR3/BST1/MMP9/PTX3/MME/SDCBP/GHDC/ORM1/TTR/CPNE1 |
| BP | GO:0042119 | neutrophil activation | 20/196 | 3.13E-07 | 0.000214 | 0.000203 | S100A8/S100A12/PYGL/MNDA/SIRPA/S100A9/FCGR2A/SIGLEC9/GCA/S100A11/LAMTOR3/BST1/MMP9/PTX3/MME/SDCBP/GHDC/ORM1/TTR/CPNE1 |
| BP | GO:0002446 | neutrophil mediated immunity | 20/196 | 3.23E-07 | 0.000214 | 0.000203 | S100A8/S100A12/PYGL/MNDA/SIRPA/S100A9/FCGR2A/SIGLEC9/GCA/S100A11/LAMTOR3/BST1/MMP9/PTX3/MME/SDCBP/GHDC/ORM1/TTR/CPNE1 |
| CC | GO:0060205 | cytoplasmic vesicle lumen | 17/200 | 6.84E-08 | 1.14E-05 | 1.09E-05 | FN1/S100A8/S100A12/PYGL/MNDA/S100A9/HBA1/FGG/SERPING1/GCA/S100A11/PTX3/SDCBP/GHDC/ORM1/TTR/SAA1 |
| CC | GO:0031983 | vesicle lumen | 17/200 | 7.14E-08 | 1.14E-05 | 1.09E-05 | FN1/S100A8/S100A12/PYGL/MNDA/S100A9/HBA1/FGG/SERPING1/GCA/S100A11/PTX3/SDCBP/GHDC/ORM1/TTR/SAA1 |
| CC | GO:0034774 | secretory granule lumen | 15/200 | 1.05E-06 | 0.000112 | 0.000107 | FN1/S100A8/S100A12/PYGL/MNDA/S100A9/FGG/SERPING1/GCA/S100A11/PTX3/SDCBP/GHDC/ORM1/TTR |
| CC | GO:0062023 | collagen-containing extracellular matrix | 15/200 | 1.80E-05 | 0.001441 | 0.001375 | FN1/LAMB2/S100A8/CCDC80/THBS2/S100A9/EFEMP1/FGG/SERPING1/MMP9/MEGF9/ORM1/SFRP2/COL6A2/SRPX |
| CC | GO:0072562 | blood microparticle | 8/200 | 0.00013 | 0.008341 | 0.007956 | FN1/CIB2/HBA1/FGG/SERPING1/IGHD/SDCBP/ORM1 |
| CC | GO:0015935 | small ribosomal subunit | 5/200 | 0.000824 | 0.043945 | 0.041921 | HBA1/MRPS18C/MRPS26/RPS26/MRPS15 |

BP: biological processes; CC: cellular components; MF: molecular functions
