# Supplementary material for: Association of Circulating, Inflammatory-Response Exosomal mRNAs With Acute Myocardial Infarction
Source: Front Cardiovasc Med. 2021 Aug 19;8:712061. doi: 10.3389/fcvm.2021.712061 (PMC8418229; doi:10.3389/fcvm.2021.712061)
Supplement: Supplementary file 5 [file Table_5.DOCX]

Table S5 Different exosomal mRNAs overlapped between the AMI and control groups and AMI and CAD

| EntrezID | logFC | p.value | adj.p.val | symbols | name |
| --- | --- | --- | --- | --- | --- |
| 10518 | -2.9545 | 1.01E-05 | 0.037459 | CIB2 | calcium and integrin binding family member 2 |
| 5079 | -2.0648 | 3.95E-06 | 0.022043 | PAX5 | paired box 5 |
| 730029 | -1.6541 | 0.004071 | 0.39733 | RPSAP19 | ribosomal protein SA pseudogene 19 |
| 100873463 | -1.3266 | 0.003113 | 0.36926 | RNA5SP202 | "RNA, 5S ribosomal pseudogene 202" |
| 6386 | 1.0433 | 0.006931 | 0.42514 | SDCBP | syndecan binding protein |
| 6648 | 1.0833 | 0.003416 | 0.37385 | SOD2 | superoxide dismutase 2 |
| 4084 | 1.1743 | 0.002824 | 0.36926 | MXD1 | MAX dimerization protein 1 |
| 79660 | 1.2294 | 0.007028 | 0.43165 | PPP1R3B | protein phosphatase 1 regulatory subunit 3B |
| 22936 | 1.2769 | 0.006923 | 0.42514 | ELL2 | elongation factor for RNA polymerase II 2 |
| 6280 | 1.3102 | 0.000873 | 0.25701 | S100A9 | S100 calcium binding protein A9 |
| 2180 | 1.3912 | 0.004452 | 0.40277 | ACSL1 | acyl-CoA synthetase long chain family member 1 |
| 23306 | 1.4012 | 0.006943 | 0.42578 | NEMP1 | nuclear envelope integral membrane protein 1 |
| 4332 | 1.4834 | 0.000592 | 0.23545 | MNDA | myeloid cell nuclear differentiation antigen |
| 353511 | 1.5343 | 0.006913 | 0.42514 | PKD1P6 | "polycystin 1, transient receptor potential channel interacting pseudogene 6" |
| 55793 | 1.5373 | 0.00656 | 0.42514 | MINDY1 | MINDY lysine 48 deubiquitinase 1 |
| 79989 | 1.5701 | 0.004486 | 0.40277 | TTC26 | tetratricopeptide repeat domain 26 |
| 5836 | 1.7185 | 0.000302 | 0.17969 | PYGL | glycogen phosphorylase L |
| 23483 | 1.7682 | 0.001503 | 0.32949 | TGDS | "TDP-glucose 4,6-dehydratase" |
| 2266 | 1.7724 | 0.001946 | 0.33114 | FGG | fibrinogen gamma chain |
| 7518 | 1.7755 | 0.005124 | 0.40658 | XRCC4 | X-ray repair cross complementing 4 |
| 646309 | 1.7763 | 0.000142 | 0.17611 | NAMPTP1 | nicotinamide phosphoribosyltransferase pseudogene 1 |
| 10135 | 1.8137 | 0.000199 | 0.17969 | NAMPT | nicotinamide phosphoribosyltransferase |
| 1401 | 1.8196 | 0.006353 | 0.42514 | CRP | C-reactive protein |
| 6948 | 1.8738 | 0.006691 | 0.42514 | TCN2 | transcobalamin 2 |
| 90326 | 1.8785 | 0.006613 | 0.42514 | THAP3 | THAP domain containing 3 |
| 23569 | 1.8786 | 0.001089 | 0.2808 | PADI4 | peptidyl arginine deiminase 4 |
| 4318 | 1.8834 | 0.003564 | 0.37583 | MMP9 | matrix metallopeptidase 9 |
| 440145 | 1.8895 | 0.005157 | 0.41708 | MZT1 | mitotic spindle organizing protein 1 |
| 6279 | 1.9146 | 9.37E-05 | 0.13066 | S100A8 | S100 calcium binding protein A8 |
| 84519 | 2.2508 | 0.002086 | 0.33791 | ACRBP | acrosin binding protein |
| 249 | 2.3123 | 0.000415 | 0.2078 | ALPL | "alkaline phosphatase, biomineralization associated" |
| 388753 | 2.3468 | 0.006376 | 0.42514 | COA6 | cytochrome c oxidase assembly factor 6 |
| 152195 | 2.4518 | 0.000298 | 0.17969 | NUDT16P1 | nudix hydrolase 16 pseudogene 1 |
| 51188 | 2.4729 | 0.002499 | 0.36705 | SS18L2 | SS18 like 2 |
| 6283 | 3.1293 | 0.000273 | 0.17969 | S100A12 | S100 calcium binding protein A12 |

logFC: log Fold Change; adj.P.Val: adjusted p value
