# Supplementary material for: Association of Circulating, Inflammatory-Response Exosomal mRNAs With Acute Myocardial Infarction
Source: Front Cardiovasc Med. 2021 Aug 19;8:712061. doi: 10.3389/fcvm.2021.712061 (PMC8418229; doi:10.3389/fcvm.2021.712061)
Supplement: Supplementary file 6 [file Table_6.DOCX]

Table S6 GO analysis of 35 different exosomal mRNAs overlapped between the AMI and control groups and AMI and CAD

| ONTOLOGY | ID | Description | Log P | Log(q-value) | Gene Ratio | Symbols |
| --- | --- | --- | --- | --- | --- | --- |
| BP | GO:0002274 | myeloid leukocyte activation | -5.99513 | -2.211125154 | 8/664 | MMP9,MNDA,PYGL,S100A8,S100A9,S100A12,SDCBP,NAMPT,FGG,SOD2,CRP,PAX5,ALPL |
| BP | GO:0002274 | myeloid leukocyte activation | -5.99513 | -2.211125154 | 8/664 | MMP9,MNDA,PYGL,S100A8,S100A9,S100A12,SDCBP,NAMPT |
| BP | GO:0010035 | response to inorganic substance | -3.23814 | -0.369001666 | 5/568 | FGG,MMP9,S100A8,SOD2,XRCC4 |
| BP | GO:0010035 | response to inorganic substance | -3.23814 | -0.369001666 | 5/568 | FGG,MMP9,S100A8,SOD2,XRCC4 |
| BP | GO:0035296 | regulation of tube diameter | -3.15774 | -0.359003658 | 3/143 | CRP,FGG,SOD2,MMP9,NAMPT,PAX5 |
| BP | GO:0035296 | regulation of tube diameter | -3.15774 | -0.359003658 | 3/143 | CRP,FGG,SOD2 |
| BP | GO:0007584 | response to nutrient | -2.62651 | -0.05110949 | 3/219 | ALPL,ACSL1,CIB2,NAMPT |
| BP | GO:0007584 | response to nutrient | -2.62651 | -0.05110949 | 3/219 | ALPL,ACSL1,CIB2 |
| BP | GO:0015980 | energy derivation by oxidation of organic compounds | -2.33558 | 0 | 3/278 | PYGL,PPP1R3B,COA6 |
| BP | GO:0015980 | energy derivation by oxidation of organic compounds | -2.33558 | 0 | 3/278 | PYGL,PPP1R3B,COA6 |

BP: biological processes
