# Supplementary material for: Association of Circulating, Inflammatory-Response Exosomal mRNAs With Acute Myocardial Infarction
Source: Front Cardiovasc Med. 2021 Aug 19;8:712061. doi: 10.3389/fcvm.2021.712061 (PMC8418229; doi:10.3389/fcvm.2021.712061)
Supplement: Supplementary file 7 [file Table_7.DOCX]

Table S7 175 exosomal mRNAs in the light-yellow module

| Gene Names | kME | kMEp | GS_Cor | GS_CorP |  | Gene Names | kME | kMEp | GS_Cor | GS_CorP |  | Gene Names | kME | kMEp | GS_Cor | GS_CorP |
| --- | --- | --- | --- | --- | --- | --- | --- | --- | --- | --- | --- | --- | --- | --- | --- | --- |
| ABL2 | 0.66 | 0.00164 | 0.420 | 0.065 |  | DNAJB1 | 0.37 | 0.109 | 0.049 | 0.839 |  | HP | 0.76 | 9.58E-05 | 0.664 | 0.001 |
| ACKR1 | 0.68 | 0.00108 | 0.256 | 0.276 |  | DNAJC5 | 0.48 | 0.0329 | 0.044 | 0.856 |  | HSD17B11 | 0.46 | 0.0423 | 0.176 | 0.458 |
| ACSL1 | 0.96 | 5.12E-11 | 0.638 | 0.002 |  | DOK3 | 0.73 | 0.000252 | 0.463 | 0.040 |  | HSPD1 | 0.70 | 0.000545 | 0.393 | 0.087 |
| ACSM3 | 0.61 | 0.00466 | 0.377 | 0.101 |  | DSTYK | 0.48 | 0.0305 | 0.180 | 0.448 |  | IFITM2 | 0.80 | 2.05E-05 | 0.581 | 0.007 |
| ADAP2 | 0.79 | 3.37E-05 | 0.261 | 0.266 |  | ELL2 | 0.89 | 1.12E-07 | 0.582 | 0.007 |  | IFRD1 | 0.86 | 1.31E-06 | 0.540 | 0.014 |
| AGO2 | 0.39 | 0.0927 | 0.334 | 0.150 |  | EMC9 | 0.88 | 3.70E-07 | 0.311 | 0.182 |  | IGFBP2 | 0.73 | 0.000239 | 0.544 | 0.013 |
| AKIRIN1 | 0.66 | 0.00168 | 0.478 | 0.033 |  | EPAS1 | 0.51 | 0.0222 | 0.136 | 0.568 |  | IRAK3 | 0.64 | 0.00237 | 0.412 | 0.071 |
| ALKBH8 | 0.72 | 0.000362 | 0.666 | 0.001 |  | ESAM | 0.47 | 0.0384 | 0.628 | 0.003 |  | IRF2BPL | 0.75 | 0.000156 | 0.376 | 0.102 |
| ALPL | 0.91 | 2.09E-08 | 0.724 | 0.000 |  | ETF1 | 0.51 | 0.0228 | 0.365 | 0.114 |  | JMJD6 | 0.82 | 1.22E-05 | 0.515 | 0.020 |
| ANKLE1 | 0.69 | 0.000737 | 0.597 | 0.005 |  | ETS2 | 0.70 | 0.000557 | 0.328 | 0.158 |  | KATNB1 | 0.54 | 0.0134 | 0.324 | 0.163 |
| ANXA5 | 0.71 | 0.000427 | 0.624 | 0.003 |  | FAM122A | 0.67 | 0.00112 | 0.239 | 0.310 |  | KCTD20 | 0.37 | 0.105 | 0.149 | 0.531 |
| AQP9 | 0.69 | 0.000808 | 0.544 | 0.013 |  | FAM129A | 0.83 | 5.98E-06 | 0.491 | 0.028 |  | KIF11 | 0.77 | 6.60E-05 | 0.543 | 0.013 |
| ARAP3 | 0.50 | 0.0239 | 0.242 | 0.304 |  | FAM212B | 0.60 | 0.00516 | 0.397 | 0.083 |  | KIF13A | 0.62 | 0.00376 | 0.422 | 0.064 |
| ARHGAP6 | 0.41 | 0.0699 | 0.486 | 0.030 |  | FCAR | 0.67 | 0.00122 | 0.321 | 0.168 |  | KIF1B | 0.47 | 0.035 | 0.330 | 0.155 |
| ARRB2 | 0.57 | 0.00944 | 0.349 | 0.132 |  | FCGR2A | 0.80 | 2.74E-05 | 0.414 | 0.070 |  | LARP7 | 0.49 | 0.027 | 0.334 | 0.150 |
| ASPSCR1 | 0.62 | 0.00371 | 0.220 | 0.351 |  | FKBP4 | 0.72 | 0.000375 | 0.145 | 0.542 |  | LDB2 | 0.58 | 0.00693 | 0.477 | 0.034 |
| ATG3 | 0.44 | 0.0519 | 0.339 | 0.144 |  | FKBP5 | 0.89 | 2.13E-07 | 0.576 | 0.008 |  | LITAF | 0.85 | 2.37E-06 | 0.659 | 0.002 |
| ATP13A3 | 0.74 | 0.000207 | 0.425 | 0.062 |  | FMNL2 | 0.57 | 0.00822 | 0.487 | 0.029 |  | LMO7 | 0.93 | 2.02E-09 | 0.479 | 0.033 |
| ATP8B3 | 0.77 | 7.00E-05 | 0.240 | 0.308 |  | FOSL2 | 0.56 | 0.01 | 0.488 | 0.029 |  | LSG1 | 0.38 | 0.0977 | 0.150 | 0.528 |
| BASP1 | 0.88 | 3.30E-07 | 0.509 | 0.022 |  | FOXM1 | 0.49 | 0.0292 | 0.110 | 0.644 |  | LYN | 0.61 | 0.00441 | 0.366 | 0.113 |
| BTN3A3 | 0.62 | 0.00335 | 0.195 | 0.410 |  | FRAT2 | 0.80 | 2.24E-05 | 0.694 | 0.001 |  | MAP3K3 | 0.58 | 0.00736 | 0.508 | 0.022 |
| CA2 | 0.46 | 0.0431 | 0.496 | 0.026 |  | FRMD4B | 0.65 | 0.00189 | 0.490 | 0.028 |  | MAPK11 | 0.76 | 0.000112 | 0.655 | 0.002 |
| CCDC68 | 0.81 | 1.61E-05 | 0.311 | 0.182 |  | GAB1 | 0.70 | 0.000577 | 0.352 | 0.128 |  | METTL9 | 0.60 | 0.00504 | 0.386 | 0.093 |
| CDC42EP4 | 0.54 | 0.0139 | -0.053 | 0.826 |  | GAS7 | 0.70 | 0.000624 | 0.561 | 0.010 |  | MITF | 0.85 | 2.72E-06 | 0.672 | 0.001 |
| CDR2L | 0.84 | 3.68E-06 | 0.371 | 0.107 |  | GBA2 | 0.84 | 3.73E-06 | 0.434 | 0.056 |  | MLKL | 0.67 | 0.00117 | 0.339 | 0.144 |
| CEP85 | 0.66 | 0.00148 | 0.402 | 0.079 |  | GCA | 0.41 | 0.0696 | 0.541 | 0.014 |  | MMP25 | 0.87 | 7.96E-07 | 0.640 | 0.002 |
| CETN3 | 0.70 | 0.000589 | 0.440 | 0.052 |  | GFOD1 | 0.54 | 0.0131 | 0.374 | 0.104 |  | MNDA | 0.70 | 0.000652 | 0.721 | 0.000 |
| CPQ | 0.64 | 0.0023 | 0.288 | 0.218 |  | GNAQ | 0.74 | 0.000205 | 0.480 | 0.032 |  | MORF4L1 | 0.49 | 0.0269 | 0.421 | 0.065 |
| CREB3 | 0.56 | 0.0103 | 0.414 | 0.070 |  | GPER1 | 0.62 | 0.00361 | 0.444 | 0.050 |  | MSL1 | 0.63 | 0.00269 | 0.489 | 0.029 |
| CRLS1 | 0.44 | 0.0519 | 0.026 | 0.914 |  | GRB2 | 0.59 | 0.00607 | 0.354 | 0.126 |  | MSRB1 | 0.48 | 0.0341 | 0.450 | 0.047 |
| CTDSP2 | 0.48 | 0.0321 | 0.158 | 0.506 |  | H3F3B | 0.73 | 0.000292 | 0.514 | 0.020 |  | MXD1 | 0.92 | 8.28E-09 | 0.688 | 0.001 |
| CTTNBP2NL | 0.68 | 0.00107 | 0.250 | 0.288 |  | HAND2 | 0.89 | 2.22E-07 | 0.317 | 0.173 |  | MYNN | 0.71 | 0.000506 | 0.293 | 0.210 |
| CXCR2 | 0.81 | 1.56E-05 | 0.762 | 0.000 |  | HECW2 | 0.42 | 0.064 | 0.297 | 0.204 |  | NAMPT | 0.90 | 8.92E-08 | 0.774 | 0.000 |
| DBF4B | 0.84 | 3.15E-06 | 0.469 | 0.037 |  | HIST1H2BG | 0.42 | 0.0678 | 0.269 | 0.251 |  | NCF2 | 0.86 | 1.06E-06 | 0.548 | 0.012 |
| NDST1 | 0.70 | 0.000548 | 0.423 | 0.063 |  | S100A8 | 0.80 | 2.15E-05 | 0.715 | 0.000 |  | ZFAND4 | 0.42 | 0.0681 | 0.229 | 0.331 |
| NDUFAF7 | 0.79 | 2.95E-05 | 0.648 | 0.002 |  | S100A9 | 0.91 | 3.49E-08 | 0.748 | 0.000 |  | ZNF467 | 0.64 | 0.00256 | 0.492 | 0.028 |
| NHSL2 | 0.47 | 0.0362 | 0.378 | 0.100 |  | SBNO2 | 0.63 | 0.00273 | 0.578 | 0.008 |  | ZNF608 | 0.85 | 1.83E-06 | 0.421 | 0.065 |
| NQO2 | 0.65 | 0.00204 | 0.318 | 0.172 |  | SDCBP | 0.76 | 0.000104 | 0.730 | 0.000 |  | VCAN | 0.66 | 0.00173 | 0.520 | 0.019 |
| NSMCE1 | 0.46 | 0.0405 | 0.067 | 0.778 |  | SGTA | 0.72 | 0.000335 | 0.511 | 0.021 |  | WASF3 | 0.49 | 0.0302 | 0.562 | 0.010 |
| OGFRL1 | 0.67 | 0.00131 | 0.716 | 0.000 |  | SH3PXD2A | 0.65 | 0.00176 | 0.631 | 0.003 |  | WDFY3 | 0.74 | 0.000206 | 0.425 | 0.062 |
| PACSIN2 | 0.57 | 0.00829 | 0.160 | 0.500 |  | SLC25A1 | 0.49 | 0.0296 | 0.247 | 0.294 |  | YLPM1 | 0.41 | 0.0761 | 0.082 | 0.731 |
| PADI2 | 0.68 | 0.000933 | 0.681 | 0.001 |  | SLC48A1 | 0.66 | 0.00149 | 0.348 | 0.133 |  | RIT1 | 0.78 | 5.40E-05 | 0.415 | 0.069 |
| PAIP2 | 0.58 | 0.00732 | 0.690 | 0.001 |  | SMIM1 | 0.57 | 0.00946 | 0.290 | 0.215 |  | RNF24 | 0.65 | 0.00179 | 0.518 | 0.019 |
| PANX2 | 0.63 | 0.00289 | 0.453 | 0.045 |  | SNX16 | 0.84 | 3.76E-06 | 0.402 | 0.079 |  | RPGRIP1L | 0.89 | 1.47E-07 | 0.461 | 0.041 |
| PDZD8 | 0.63 | 0.00279 | 0.634 | 0.003 |  | SOCS1 | 0.85 | 2.20E-06 | 0.436 | 0.055 |  | S100A12 | 0.74 | 0.000171 | 0.658 | 0.002 |
| PELI1 | 0.60 | 0.0053 | 0.120 | 0.614 |  | SOD2 | 0.93 | 3.86E-09 | 0.735 | 0.000 |  | RC3H1 | 0.59 | 0.00658 | 0.306 | 0.189 |
| PFKFB3 | 0.83 | 6.60E-06 | 0.380 | 0.098 |  | SPI1 | 0.71 | 0.000483 | 0.574 | 0.008 |  | RELB | 0.68 | 0.00103 | 0.480 | 0.032 |
| PGD | 0.56 | 0.0107 | 0.548 | 0.012 |  | SREBF1 | 0.33 | 0.162 | -0.069 | 0.774 |  | RGS2 | 0.42 | 0.0686 | 0.640 | 0.002 |
| PHC2 | 0.49 | 0.0276 | 0.610 | 0.004 |  | SREK1IP1 | 0.68 | 0.000961 | 0.486 | 0.030 |  | RHOG | 0.60 | 0.00541 | 0.478 | 0.033 |
| PIGS | 0.59 | 0.0065 | 0.247 | 0.294 |  | STK24 | 0.63 | 0.00304 | 0.389 | 0.090 |  | TULP3 | 0.68 | 0.00102 | 0.443 | 0.050 |
| PNPLA2 | 0.50 | 0.0245 | 0.021 | 0.930 |  | STX3 | 0.40 | 0.0797 | 0.249 | 0.290 |  | UBR3 | 0.62 | 0.00366 | 0.257 | 0.274 |
| PNPLA6 | 0.41 | 0.0746 | 0.069 | 0.773 |  | SULT1B1 | 0.90 | 8.42E-08 | 0.444 | 0.050 |  | UIMC1 | 0.67 | 0.0012 | 0.302 | 0.196 |
| POR | 0.55 | 0.0122 | 0.056 | 0.815 |  | SUPT7L | 0.83 | 6.88E-06 | 0.401 | 0.080 |  | USP40 | 0.53 | 0.0168 | 0.148 | 0.533 |
| PPP1R18 | 0.79 | 3.31E-05 | 0.464 | 0.039 |  | SVIL | 0.79 | 2.95E-05 | 0.323 | 0.165 |  | RAB7A | 0.56 | 0.0104 | 0.367 | 0.111 |
| PPP1R3B | 0.61 | 0.00453 | 0.695 | 0.001 |  | TBC1D1 | 0.55 | 0.0116 | 0.210 | 0.374 |  | RALB | 0.69 | 0.000764 | 0.350 | 0.130 |
| PROK2 | 0.85 | 2.09E-06 | 0.522 | 0.018 |  | TEAD2 | 0.83 | 6.00E-06 | 0.327 | 0.159 |  | TNFRSF10C | 0.82 | 1.13E-05 | 0.536 | 0.015 |
| PYGL | 0.93 | 3.70E-09 | 0.654 | 0.002 |  | TFG | 0.67 | 0.00122 | 0.563 | 0.010 |  | TRIP13 | 0.69 | 0.000839 | 0.311 | 0.182 |
| RAB3C | 0.61 | 0.00403 | 0.233 | 0.323 |  | TJP2 | 0.69 | 0.000705 | 0.413 | 0.070 |  |  |  |  |  |  |
| RAB3D | 0.53 | 0.0175 | 0.360 | 0.119 |  | TMEM131 | 0.69 | 0.000743 | 0.277 | 0.237 |  |  |  |  |  |  |

kME: K-means; kMEp: K-means p value; GS_Cor: gene significance correlation; GS_Cor P: gene significance correlation p value.
